# Novel ginsenoside derivative 20(*S*)-Rh2E2 suppresses tumor growth and metastasis in vivo and in vitro via intervention of cancer cell energy metabolism

**DOI:** 10.1038/s41419-020-02881-4

**Published:** 2020-08-14

**Authors:** Qi Huang, Hui Zhang, Li Ping Bai, Betty Yuen Kwan Law, Haoming Xiong, Xiaobo Zhou, Riping Xiao, Yuan Qing Qu, Simon Wing Fai Mok, Liang Liu, Vincent Kam Wai Wong

**Affiliations:** State Key Laboratory of Quality Research in Chinese Medicine, Macau University of Science and Technology, Macau, China

**Keywords:** Drug development, Pharmacodynamics

## Abstract

Increased energy metabolism is responsible for supporting the abnormally upregulated proliferation and biosynthesis of cancer cells. The key cellular energy sensor AMP-activated protein kinase (AMPK) and the glycolytic enzyme alpha-enolase (α-enolase) have been identified as the targets for active components of ginseng. Accordingly, ginseng or ginsenosides have been demonstrated with their potential values for the treatment and/or prevention of cancer *via* the regulation of energy balance. Notably, our previous study demonstrated that the *R*-form derivative of 20(*R*)-Rh2, 20(*R*)-Rh2E2 exhibits specific and potent anti-tumor effect via suppression of cancer energy metabolism. However, the uncertain pharmacological effect of *S*-form derivative, 20(*S*)-Rh2E2, the by-product during the synthesis of 20(*R*)-Rh2E2 from parental compound 20(*R/S*)-Rh2 (with both *R*- and *S*-form), retarded the industrialized production, research and development of this novel effective candidate drug. In this study, 20(*S*)-Rh2E2 was structurally modified from pure 20(*S*)-Rh2, and this novel compound was directly compared with 20(*R*)-Rh2E2 for their in vitro and in vivo antitumor efficacy. Results showed that 20(*S*)-Rh2E2 effectively inhibited tumor growth and metastasis in a lung xenograft mouse model. Most importantly, animal administrated with 20(*S*)-Rh2E2 up to 320 mg/kg/day survived with no significant body weight lost or observable toxicity upon 7-day treatment. In addition, we revealed that 20(*S*)-Rh2E2 specifically suppressed cancer cell energy metabolism via the downregulation of metabolic enzyme α-enolase, leading to the reduction of lactate, acetyl-coenzyme (acetyl CoA) and adenosine triphosphate (ATP) production in Lewis lung cancer cells (LLC-1), but not normal cells. These findings are consistent to the results obtained from previous studies using a similar isomer 20(*R*)-Rh2E2. Collectively, current results suggested that 20(*R*/*S*)-Rh2E2 isomers could be the new and safe anti-metabolic agents by acting as the tumor metabolic suppressors, which could be generated from 20(*R/S*)-Rh2 in industrialized scale with low cost.

## Introduction

Lung cancer is the most prevalent cancer type and the leading cause of cancer death globally^1,2^. Natural compounds derived from Chinese herbal medicines with polypharmacological property may overcome poor prognosis and serious side effects^3–5^, amongst which, ginseng or its active components have been studied extensively in the recent decade. Rg3 and Rh2, are the main bioactive constituents of ginseng exhibiting profound anti-cancer effects^6^. However, ginsenosides generally demonstrate poor solubility and cytotoxic non-specificity towards cancer cells^7,8^. Notably, structurally modified ginsenosides with optimized chemical stability and biological activity per se representing a potential medicinal alternative accordingly^9^.

The current study revealed that Rh2 exists as two stereoisomeric forms, 20(*S*)- and 20(*R*)-Rh2 (ref. ^10^), with different spatial structure of the hydroxyl group at chiral carbon-20 center^11^. Owing to the low bioavailability of Rh2 (ref. ^12^), we have modified the chemical structure of 20(*R*)-Rh2 to a newly synthesized ginsenoside derivative called 20(*R*)-Rh2E2 with improved solubility and higher anticancer potency when compared with Rg3 (ref. ^13^). Unlike its parental precursor, 20(*R*)-Rh2E2 shows tumor specific in the LLC-1 cancer cells, and exhibits no cytotoxicity towards normal lung fibroblasts, which also suppresses the tumor growth in a mice xenografted with lung cancer cells showing no adverse effect^13^. In addition, 20(*R*)-Rh2E2 specifically suppresses the energy-based metabolism of LLC-1 cells *via* the inhibition of mitochondrial enzymes and arrest in cell cycle S-phase^13^. However, 20(*S*)-Rh2E2 with uncertain pharmacological effects is the byproduct exist during the synthesis process of 20(*R*)-Rh2E2 unless 20(*R*)-Rh2 is firstly purified from the 50:50 racemic mixtures of 20(*R/S*)-Rh2, which renders the further development of the compound.

In fact, the chiral nature of compounds has been a significant concern for centuries^14^. In contrast to most medicinal compounds exploited from natural source, they occur in their optical active conformations as either the (*R*) or (*S*) enantiomer, and the synthetic compounds of desired are commonly mixed up with other nonsuperimposable mirror-image structures upon chemical synthesis^15^. The pharmacological behaviors of the different stereochemical configurations of a particular compound could be varied^16^. For Rh2, 20(*S*)-Rh2 reduced the viability of human colorectal cancer cells by inhibiting the interleukin-6 (IL-6)-induced activation of signal transducer and transcriptional activator 3 (STAT3) pathway and the expression of matrix metalloproteinases (MMPs), with such pharmacological activities much stronger than that of 20(*R*)-Rh2 (ref. ^17^). In addition, 20(*S*)-Rh2 appeared to target a more board spectrum of cancers, while 20(*R*)-Rh2 did not inhibit their growth at various concentration ranges^18^. It is worth mentioning that 20(*S*)-Rh2 is cost less compared with 20(*R*)-Rh2.

Therefore, it is important to validate the therapeutic potential and cytotoxicity of 20(*S*)-Rh2E2. In this study, the potent anti-cancer effect of 20(*S*)-Rh2E2 were confirmed with no significant toxicity observed. The solubility of 20(*S*)-Rh2E2 was significantly improved when compared with the precursor 20(*S*)-Rh2. Similar to the (*R*) enantiomer, 20(*S*)-Rh2E2 suppressed the expression of oncogenic proteins for cell invasion, metastasis, proliferation, and cell cycle progression in LLC-1 cells. In addition, the modulation of energy-associated machineries, including mitochondrial aerobic oxidation, fatty acid β-oxidation, AMPK energy-sensing pathway, and glycolysis were analyzed. Intriguingly, we unraveled that 20(*S*)-Rh2E2 suppressed all the energy production pathways specifically in LLC-1 cells, without affecting the normal counterpart. Therefore, 20(*S*)-Rh2E2 could be a valuable therapeutic inhibitor of cancer cell metabolism.

## Results

### 20(*S*)-Rh2E2 suppresses LLC-1 tumor growth and metastasis in xenograft model of C57BL/6 mice

**Fig. 1:**
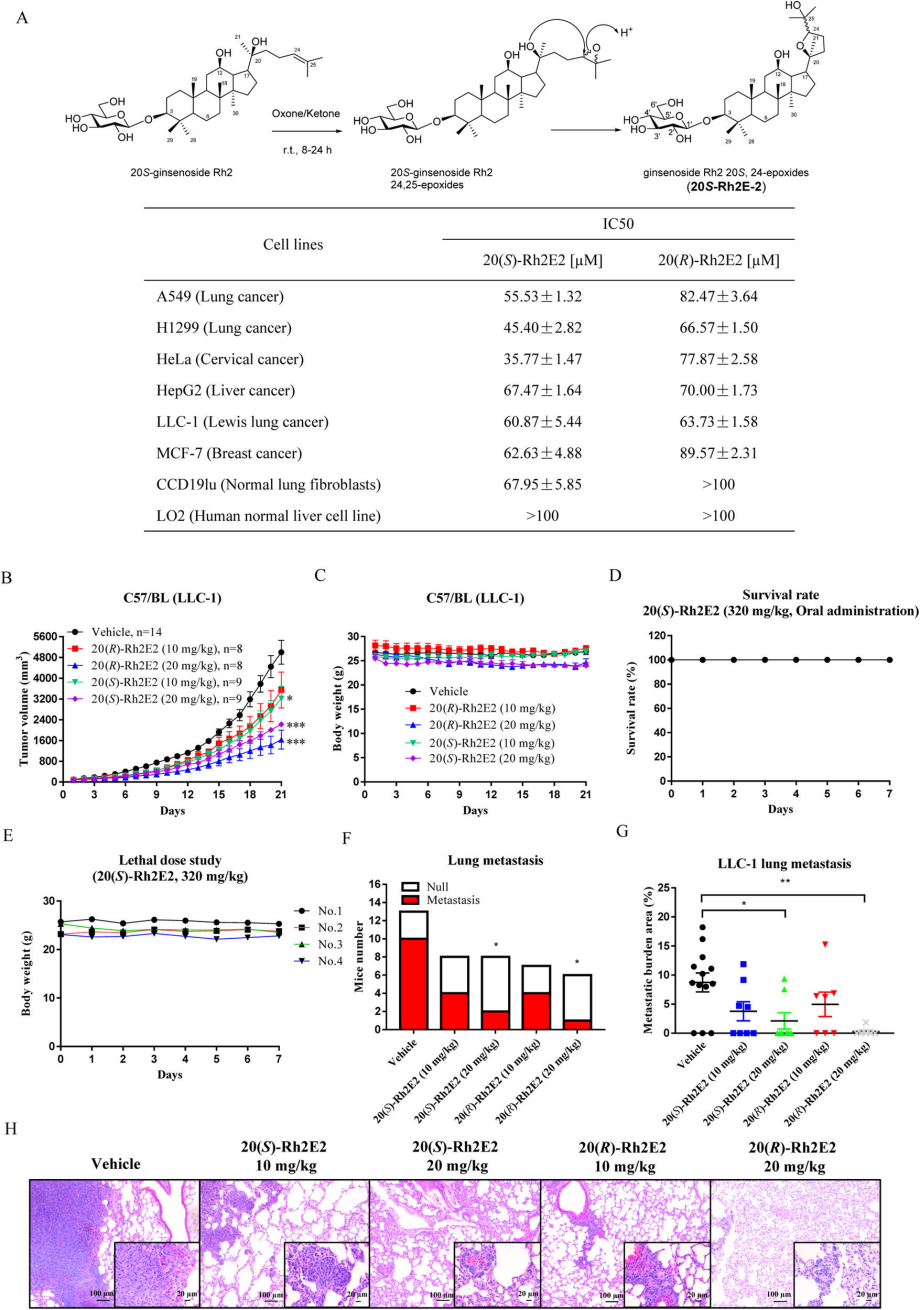
20(*S*)-Rh2E2 suppresses LLC-1 tumor growth and metastasis in LLC-1 bearing C57BL/6 mouse model. **a**: Organic synthesis of 20(*S*)-Rh2E2 and IC_50_ values of 20(*S*)/(*R*)-Rh2E2 on normal lung fibroblasts (CCD19Lu), human normal hepatocytes (LO2) and other cancer cells. Cellular cytotoxicity was measured by MTT assay after 72 h incubation. Representative results were shown as mean ± S.D. from three independent experiments. **b**, **c** LLC-1 bearing C57BL/6 mice treated with 20(*S*)-Rh2E2 [10 or 20 mg/kg/day] or 20(*R*)-Rh2E2 [10 or 20 mg/kg/day] via IP injection for 21 days. Consecutive treatment of 20(*S*)-Rh2E2 and 20(*R*)-Rh2E2 enhanced tumor growth inhibition. Mice tumor volume monitorization and mice body weight are displayed respectively. All data represent mean ± SEM. *P < 0.05, **P < 0.01, ***P < 0.001 compared to the vehicle-treated group, Student’s *t*-test. **d**, **e** Sub-chronic lethal dose treatment of 20(*S*)-Rh2E2. C57BL/6 mice were orally administrated with 320 mg/kg of 20(*S*)-Rh2E2 for 7-days. The survival rate of mice and their body weight were monitored every day. **f** 20(*S*)-Rh2E2 and 20(*R*)-Rh2E2 suppressed the lung tumor metastasis in LLC-1 bearing mice xenograft. The bar chart represents the number of mice with lung metastatic lesions (red area) and the number of mice without metastatic lesion (white area) are shown. *P < 0.05, **P < 0.01, ***P < 0.001, Chi-square test. **g**, **h** 20(*S*)-Rh2E2 and 20(*R*)-Rh2E2 reduced the metastatic burden area. The percentage of metastatic burden area is displayed in (**g**). Each dot represents the mice with particular metastatic burden area. Data represent mean ± SEM. *P < 0.05, **P < 0.01, ***P <0.001, Mann–Whitney *t*-test. **h** Representative H&E stained lung sections images with metastatic lesions. Infiltrating metastatic cells are visualized in higher magnification. Scale bar = 1 mm (×2.5), scale bar = 100 μm (×10), and scale bar = 20 μm (×40).

Our previous studies demonstrated that 20(*R*)-Rh2E2 with enhanced solubility could render the new compound with cancer-specific cytotoxicity^13^. Of note, 20(*S*)-Rh2 also demonstrated poor solubility, however, a relatively stronger cellular cytotoxicity when compared with 20(*R*)-Rh2 (ref. ^19^). Accordingly, we structurally modified the 20(*S*)-Rh2 to a newly synthesized Rh2 derivative called 20(*S*)-Rh2E2 (Fig. 1a) for assessing its cytotoxic effects. As shown in Fig. 1a and Supplementary Fig. S1, 20(*S*)-Rh2E2 exhibited cytotoxic effects on all tested cancer. Moreover, the cytotoxicity towards the normal lung fibroblasts CCD19Lu is 67.95 ± 5.85 μM, while 20(*S*)-Rh2E2 showed little or no cytotoxic effect on human normal hepatocytes LO2 at dosage over 100 μM, suggesting the differential sensitivity of compounds among the human normal cells. In contrast, 20(*R*)-Rh2E2 induced specific cytotoxicity towards all the cancer cell lines, and in line with previous findings^13^.

To further confirm the potential anti-tumor effects of 20(*S*)-Rh2E2, a mouse lung cancer LLC-1 xenograft model was established using C57BL/6 mice. As shown in Fig. 1b, IP injection of 20(*S*)-Rh2E2 at 10 and 20 mg/kg/day demonstrated dose-dependent inhibition of tumor growth up to 35.62% (*P* < 0.05) and 55.49% (*P* < 0.001) compared to control group, respectively. While the tumor growth in mice receiving 20 mg/kg/day of 20(*R*)-Rh2E2 demonstrated an inhibition of tumor growth up to 67.28% (*P* < 0.001). In addition, treatment with 20(*S*)- or 20(*R*)-Rh2E2 showed no significant reduction in body weight and vital organs, suggesting the non-toxic property of 20(*S*)- and 20(*R*)-Rh2E2 in vivo (Fig. 1c and Supplementary Fig. S2). The therapeutic safety window of 20(*S*)-Rh2E2 was further evaluated by oral administration at its sub-chronic lethal dose. Notably, no harmful effect to the treated animals was observed up to 320 mg/kg/day of 20(*S*)-Rh2E2, all the animals survived with no decline in body weight after the course of 7-day treatment (Fig. 1d, e).

Tumor metastasis is the major cause of death in lung cancer patients, lung tissues from the vehicle- and 20(*S*)-Rh2E2- or 20(*R*)-Rh2E2-treated mice at 10 and 20 mg/kg were analyzed in hematoxylin and eosin (H&E) staining. As shown in Fig. 1f, the number of mice with lung metastasis was markedly reduced in treatment groups of high dosage of 20(*S*)- and 20(*R*)-Rh2E2 compared to vehicle. In addition, the average of metastatic burden area per group was 8.74% for vehicle control, 3.78% for 20(*S*)-Rh2E2 (10 mg/kg), 2.11% for 20(*S*)-Rh2E2 (20 mg/kg), 4.97% for 20(*R*)-Rh2E2 (10 mg/kg) and 0.31% for 20(*R*)-Rh2E2 (20 mg/kg), respectively (Fig. 1g). As shown in Fig. 1h, the staining of the lung tissues in the vehicle-treated control group were more intense, and the stained LLC-1 cells were larger than the normal lung fibroblasts, suggesting the metastasis of the inoculated LLC-1 cells from the subcutaneous dorsal region to lung tissues. Taken together, 20(*S*)-Rh2E2 could inhibit the tumor growth and metastasis of LLC-1 bearing mice with similar anti-tumor potency of 20(*R*)-Rh2E2.

### 20(*S*)-Rh2E2 suppresses the expression of metastatic markers α-enolase or stathmin in vitro and in vivo

**Fig. 2:**
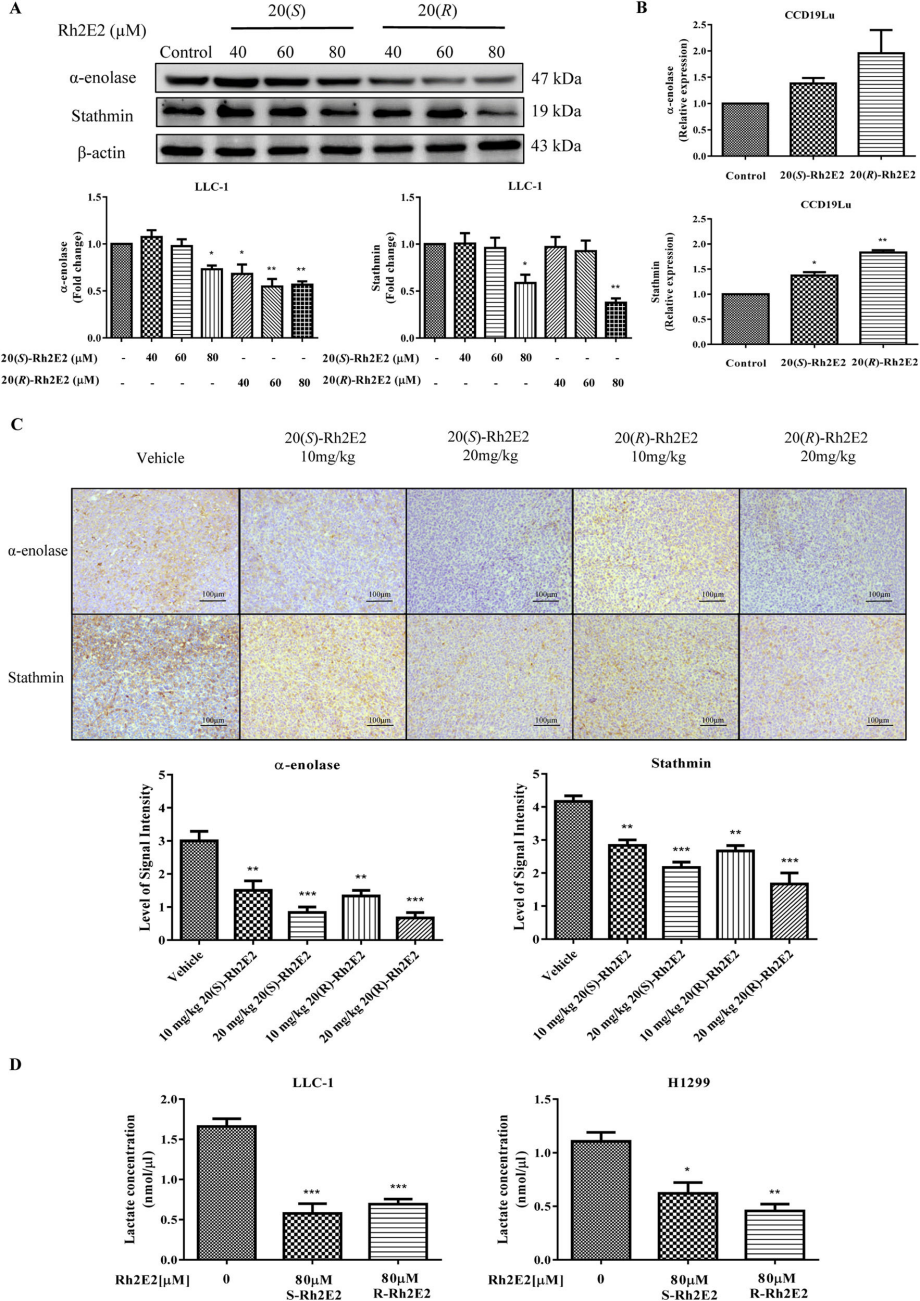
20(*S*)-Rh2E2 suppresses the expression of α-enolase or stathmin in LLC-1 cancer cells and tumor tissue dissected from LLC-1 xenograft model. **a** Both α-enolase and stathmin were suppressed after 20(*S*)-Rh2E2 and 20(*R*)-Rh2E2 treatment. Representative immunoblots and the protein quantification were shown from three independent experiments; **P < 0.01, ***P < 0.001, one-way ANOVA analysis. **b** Quantification of metastatic markers α-enolase and stathmin genes expression in 20(*S*)*/*(*R*)-Rh2E2-treated CCD19Lu cells by RT-qPCR. **c** 20(*S*)-Rh2E2 and 20(*R*)-Rh2E2 suppressed the expression of α-enolase and stathmin in tumor tissues harvested from LLC-1 xenograft mice. α-enolase and stathmin staining images were representative of 5 tumor sections from 6 animals of each group. The level of signal intensity was scored from 1–5 (5 is maximum) and took the average from five different views of each section taken in ×20 magnifications. **d** 20(*S*)-Rh2E2 and 20(*R*)-Rh2E2 suppressed the release of lactate in LLC-1 and H1299 cancer cells. *P < 0.05, **P < 0.01, ***P < 0.001, one-way ANOVA analysis.

The metastasis of cancer is associated with the expression of proteins such as α-enolase, stathmin, cofilin-1, Rho GDP-dissociation inhibitor 1 and thromboxane-A synthase, which serve as potential prognostic markers^20–24^ and may participate in the anti-metastatic actions of 20(*S*/*R*)-Rh2E2. Notably, our previous studies revealed the crucial role of α-enolase and stathmin in 20(*R*)-Rh2E2-inhibited cancer cells invasion^13^. As such, the cellular expression profile of α-enolase and stathmin in LLC-1 and CCD19Lu were further examined after the treatment of 20(*S*)-Rh2E2. As shown in Fig. 2a and Supplementary Fig. S3, the expression of α-enolase and stathmin were dose-dependently suppressed by the administration of either 20(*S*)- or 20(*R*)-Rh2E2 when compared to untreated control in LLC-1 cells. However, 20(*S*)-Rh2E2, which was more effective in reduction of cell viability, has a weaker downregulation effect on these markers. Unfortunately, we cannot detect any protein expression of either α-enolase or stathmin in CCD19lu normal cells. Alternatively, the expression levels of these two genes were further validated individually by Real-Time quantitative PCR (RT-qPCR) using gene specific primers (Supplementary Table S1). As shown in Fig. 2b, the treatment of 20(*S*)- and 20(*R*)-Rh2E2 showed upregulation trend in the gene expression of α-enolase or stathmin in CCD19Lu cells. In addition, immunohistochemical analysis of tumor tissues collected from LLC-1 xenograft model treated with either 20(*S*)- or 20(*R*)-Rh2E2 showed similar expression signal, in which, the expression of α-enolase and stathmin were significantly suppressed after the treatment of 20(*S*)- or 20(*R*)-Rh2E2 (Fig. 2c). Since high levels of lactic acid are beneficial to the acidic tumor microenvironment and promote the metastasis and invasion of cancer^25^. We accordingly determined the lactate secretion in 20(*S*)*/*(*R*)-Rh2E2-treated cancer cells. As expected, the results showed that lactate secretion was downregulated in LLC-1 and H1299 cancer cells upon the treatment of 20(*S*)- and 20(*R*)-Rh2E2 (Fig. 2d). These findings were coincided with the in vivo anti-metastatic effect of 20(*S*)/(*R*)-Rh2E2 in LLC-1 bearing mice shown in Fig. 1f–h.

### 20(*S*)-Rh2E2 dose-dependently inhibits the cell migration and cell invasion ability of H1299 lung cancer cells

**Fig. 3:**
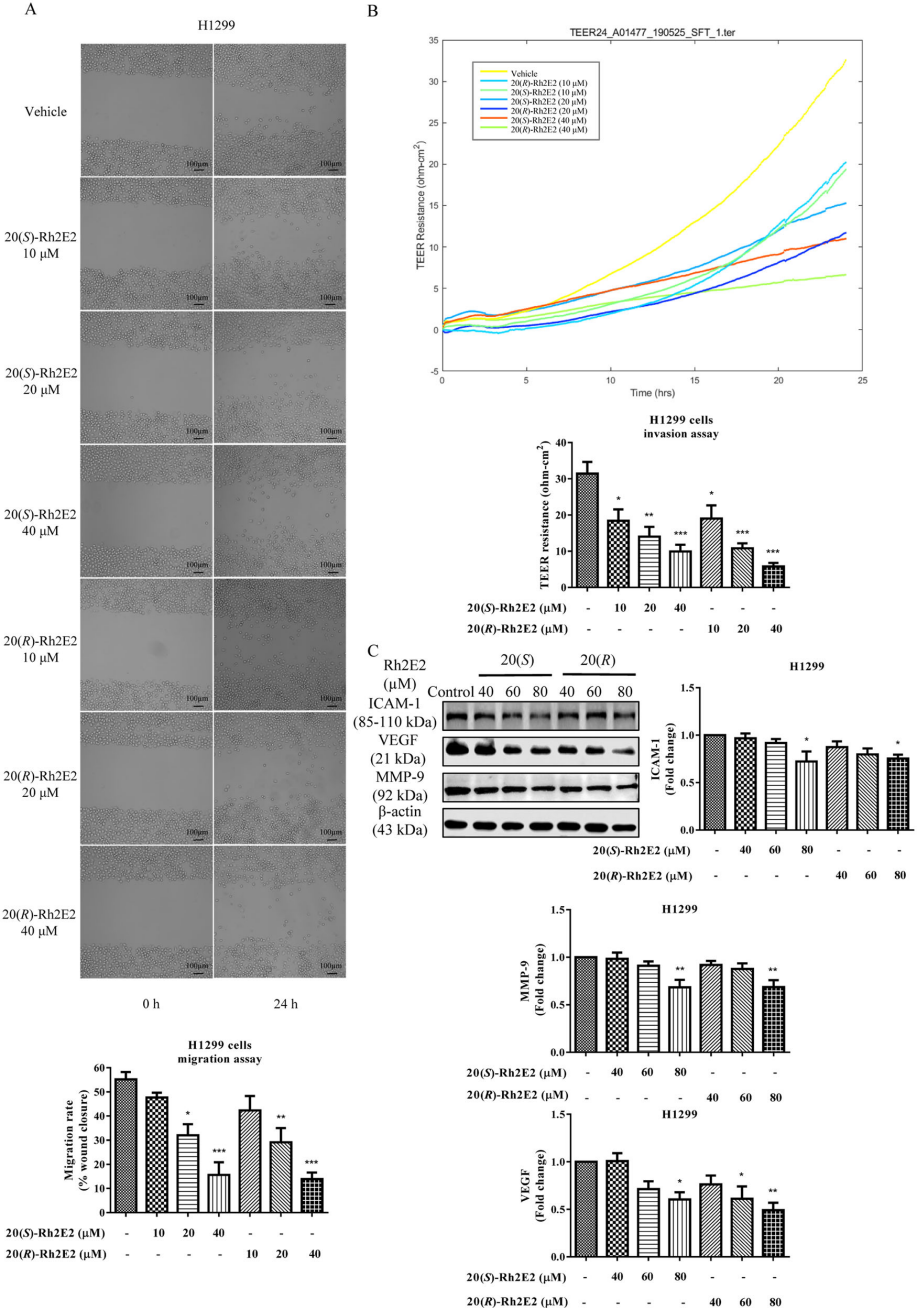
20(*S*)-Rh2E2 dose-dependently inhibits the cell migration and cell invasion ability of H1299. **a** 20(*S*)-Rh2E2 and 20(*R*)-Rh2E2 dose-dependently inhibited the cell migration ability of H1299 lung cancer cells. Wound closure was monitored by visual examination at the indicated time points using an inverted bright field microscope with ×10 magnifications. **b** 20(*S*)-Rh2E2 and 20(*R*)-Rh2E2 dose-dependently inhibited the cell invasion ability of H1299 lung cancer cells. Data were collected continuously and reported as real-time changes in barrier function of cell layers in ohm-cm^2^ by TEER24 system. **c** 20(*S*)-Rh2E2 and 20(*R*)-Rh2E2 suppressed the expression of markers for cell adhesion, invasion and angiogenesis including intercellular adhesion molecule-1 (ICAM-1), vascular endothelial growth factor (VEGF) and MMP-9. H1299 cells were treated with 20(*S*)/(*R*)-Rh2E2 (40 µM, 60 µM, 80 µM) for 24 h, respectively. Representative immunoblots and the protein quantification are shown; mean ± S.D., *n* = 3; *P < 0.05, **P < 0.01, one-way ANOVA analysis.

20(*R*)-Rh2E2 has been demonstrated to inhibit cancer cell invasion by suppression of α-enolase or stathmin^13^. As 20(*S*)-Rh2E2 was confirmed to downregulate the expression of α-enolase or stathmin (Fig. 2b), we therefore examined and compared the migration and invasion ability of H1299 human lung cancer cells between 20(*S*)- and 20(*R*)-Rh2E2. As shown in Fig. 3a, 20(*S*)/(*R*)-Rh2E2 could suppress the migration of H1299 cells upon treatment at sub-lethal doses. Besides, transepithelial electrical resistance (TEER) resistance-based cell invasion assessment further indicated that both compounds could dose-dependently inhibit the cell invasion ability of H1299 (Fig. 3b). Interestingly, the protein markers for cell adhesion, cell invasion, and angiogenesis were concomitantly downregulated upon the treatment of 20(*S*)- or 20(*R*)-Rh2E2 (Fig. 3c and Supplementary Fig. S4). Moreover, a reduction of the expression of invasive factor VEGF after the treatment was associated with the findings in Fig. 2d where lactate secretion was downregulated in 20(*S*)*/*(*R*)-Rh2E2-treated LLC-1 and H1299 cancer cells. These findings revealed that 20(*S*)-Rh2E2 coincided with 20(*R*)-Rh2E2, in which both compounds could inhibit the metastasis of cancer cells via the manipulation of migratory and invasion signaling.

### 20(*S*)-Rh2E2 specifically suppresses cancer cell energy metabolism via inhibition of mitochondrial respiration and glycolysis-associated metabolic enzymes

**Fig. 4:**
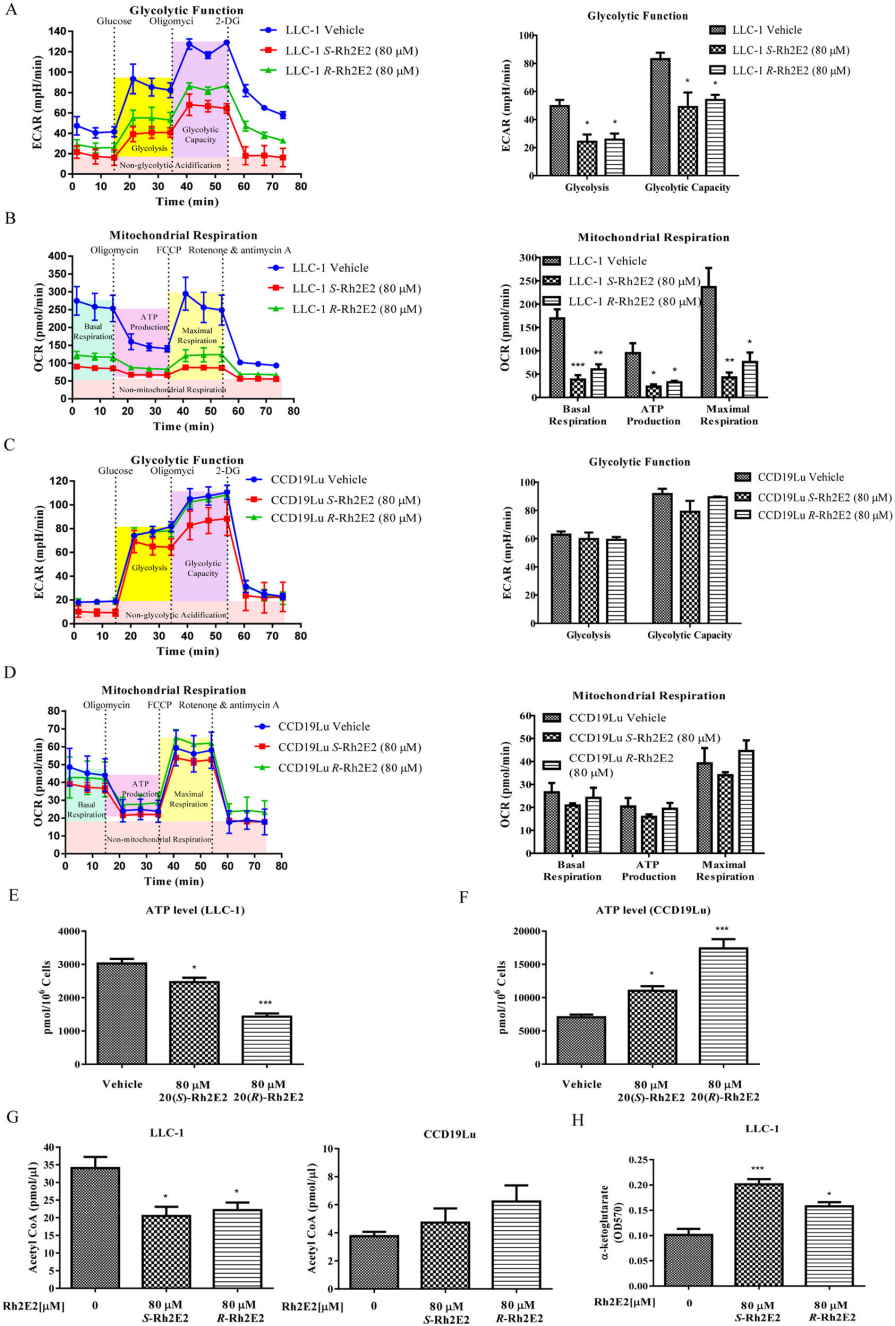
Effect of 20(*S*)-Rh2E2 on the metabolic reprograming of LLC-1 cancer cells. **a** The comparison of glycolytic profile of LLC-1 cells upon the treatment of 20(*S*)- or 20(*R)*-Rh2E2. The glycolytic function was measured by directly detecting the ECAR of cells. The compounds (glucose, oligomycin, 2-DG) were serially injected to measure the glycolysis and glycolytic capacity, respectively. **b** The comparison of mitochondrial respiration profile of LLC-1 cells upon the treatment of 20(*S*)- or 20(*R)*-Rh2E2. Mitochondrial respiration test determines the key parameters of mitochondrial function by directly measuring the OCR of cells. The compounds (oligomycin, FCCP, and a mix of rotenone and antimycin A) were serially injected to measure the basal respiration, ATP production, and maximal respiration, respectively. **c** The comparison of glycolytic profile of CCD19Lu cells upon the treatment of 20(*S*)- or 20(*R*)-Rh2E2. **d** The comparison of mitochondrial respiration profile of LLC-1 cells upon the treatment of 20(*S*)- or 20(*R)*-Rh2E2. **e** 20(*S*)-Rh2E2 and 20(*R*)-Rh2E2 decreased the ATP production in LLC-1 cancer cells. **f** 20(*S*)-Rh2E2 and 20(*R*)-Rh2E2 enhanced the ATP generation in CCD19Lu normal cells. The amount of energy metabolites was calculated as pmol/10^6^ cells. **g** 20(*S*)-Rh2E2 and 20(*R*)-Rh2E2 specifically reduced the production of acetyl CoA in LLC-1 lung cancer cells, but not CCD19Lu normal cells. **h** 20(*S*)-Rh2E2 and 20(*R*)-Rh2E2 enhanced the accumulation of α-ketoglutarate, the energy metabolite of TCA cycle in LLC-1 cells.

Unrestricted growth of tumor cells are associated with the upregulation of glucose metabolism and the overexpression of glycolytic enzymes, which support the abnormal proliferation and expansion of tumors^26^. We have demonstrated the inhibitory effect of 20(*R*)-Rh2E2 on glycolytic enzyme α-enolase, thereby contributed to the decrease of glycolysis and energy production^13^. Therefore, seahorse analysis was used to assess and compare the effect of 20(*S*)-Rh2E2 with 20(*R*)-Rh2E2 on the extracellular acidification rate (ECAR) and oxygen consumption rate (OCR) in LLC-1 and CCD19Lu cells. As shown in Fig. 4a, the basal glycolysis and glycolytic capacity were suppressed upon the treatment of 20(*S*)- or 20(*R*)-Rh2E2 in LLC-1 cells. Concomitantly, the cellular basal respiration, ATP production, and maximal respiration were all decreased in 20(*S*)/(*R*)-Rh2E2-treated LLC-1 cells (Fig. 4b). In contrast, none of these two compounds would cause inhibitory effects on the basal glycolysis, glycolytic capacity, and mitochondrial respiration in normal lung fibroblasts, CCD19lu compared to vehicle (Fig. 4c, d), suggesting the tumor specific of 20(*S*)*/*(*R*)-Rh2E2 in targeting cell energy metabolism. Dysregulation of metabolic enzymes involved in oxidative phosphorylation can lead to mitochondrial respiratory dysfunction and energy metabolism failure^27^. We, therefore, determined the expression of these metabolic enzymes in cancer cells in response to 20(*S*)*/*(*R*)-Rh2E2 treatment. The expression levels of genes were validated by RT-qPCR using primers with sequence as listed in Supplementary Table S2. As shown in Supplementary Fig. S5, the genes encoding the metabolic enzymes for oxidation phosphorylation such as Atp5l, Cox4i1, Cox5a, Cox7a2, Ndufc2, Ndufs4, Sdhd, Uqcrq^28^ were significantly downregulated in 20(*S*)*/*(*R*)-Rh2E2-treated LLC-1 cancer cells, but not in normal cells. These findings were coincided with the effect of these compounds in mitochondrial respiratory capacity in cancer and normal cells. It has been suggested that changes in the amount of synthetic mitochondrial DNA can result in mutations, deletions, or reduced synthesis of mitochondria^29,30^. Accordingly, we also designed the specific primers for detection of mitochondrial DNA level, such as Top1mt, Polg, Dab1. The expression levels of these 3 genes were validated by RT-qPCR using gene-specific primers (Supplementary Table S3). As shown in Supplementary Fig. S6, the levels of these genes were markedly suppressed in 20(*S*)*/*(*R*)-Rh2E2-treated LLC-1 cancer cells, suggesting that the number of mitochondria in cancer cells could be reduced upon 20(*S*)*/*(*R*)-Rh2E2 treatment, whereas some of these markers were upregulated in CCD19lu cells. Given the cancer cells adopt the glycolytic pathway, which yields less ATP than the mitochondrial aerobic oxidation utilized by normal cells^31^, here, we showed that an equal number of LLC-1 cells would generate less ATP than that of the normal cells (Fig. 4e, f). As expected, 20(*S*)*/*(*R*)-Rh2E2-treated LLC-1 cells showed a decrease in ATP production (Fig. 4e). However, treatment of either 20(*S*)- or 20(*R*)-Rh2E2 in human normal lung fibroblasts could effectively enhance the level of ATP production (Fig. 4f), which findings suggested that 20(*S*)- and 20(*R*)-Rh2E2 exhibit a biphasic effect on energy production between normal and cancer cells. We further addressed whether 20(*S*)-Rh2E2 treatment suppresses the key metabolic enzymes of mitochondria. Several key fatty acid β-oxidation enzymes have been downregulated by 20(*R*)-Rh2E2 (ref. ^13^), we, therefore, validated the effect of 20(*S*)-Rh2E2 in regulation of metabolic enzymes in fatty acid β-oxidation by examining their end product, acetyl-CoA. A reduction in acetyl-CoA was observed in either 20(*S*)-Rh2E2- or 20(*R*)-Rh2E2-treated LLC-1 cells, but not in CCD19Lu normal lung fibroblasts (Fig. 4g), suggesting that neither the metabolic enzymes nor acetyl-CoA were affected in 20(*S*)*/*(*R*)-Rh2E2-treated normal cells. On the other hand, the level of metabolic enzymes involved in the tricarboxylic acid cycle (TCA) cycle^32^, have been modulated by 20(*R*)-Rh2E2 (ref. ^13^). Of note, upregulation of aconitase together with downregulation of α-ketoglutarate (α-KG) dehydrogenase led to the accumulation of the TCA cycle intermediate, α-KG^33^. Our results indicated that either form of Rh2E2 significantly increased α-KG in LLC-1 cells (Fig. 4h), indicating that the TCA cycle was retarded upon 20(*S*)*/*(*R*)-Rh2E2 treatment. Collectively, these findings suggest that 20(*S*)-Rh2E2 coincided with 20(*R*)-Rh2E2 specifically suppress cell metabolism and energy production in cancer cells, but not in normal cells.

### 20(*S*)-Rh2E2 induces S-phase cell cycle arrest in LLC-1 cancer cells through downregulation of S-phase specific cyclin-dependent kinases (Cdks)/Cyclins expression

**Fig. 5:**
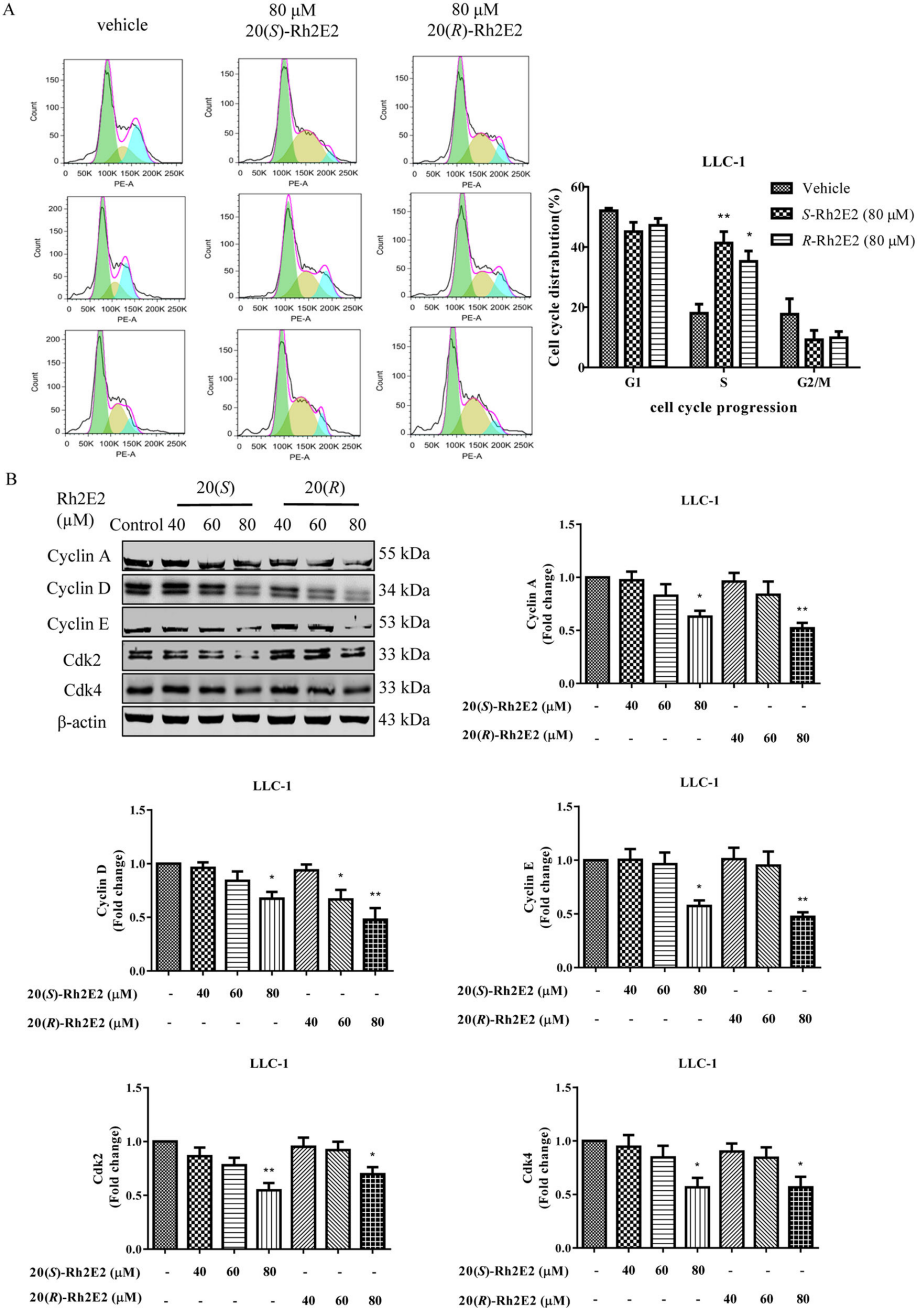
20(*S*)-Rh2E2 induces S-phase cell cycle arrest through regulation of Cdks/Cyclins and Cdks inhibitors expression. **a** Effects of 20(*S*)-Rh2E2 and 20(*R*)-Rh2E2 on cell cycle progression in LLC-1 cancer cells. Exponentially growing LLC-1 cells were synchronized in serum-free medium for 24 h. Then the cells were incubated with the 80 µM 20(*S*)-Rh2E2 or 80 µM 20(*R*)-Rh2E2 for 48 h. The cell cycle progression was evaluated using propidium iodide staining and flow cytometry analysis. The bar chart indicated the results of quantitative analysis of cell-cycle distribution (% of cell population). Mean ± SEM were from three independent experiments (One-way ANOVA: *P < 0.05, **P < 0.01). **b** Effect of 20(*S*)-Rh2E2 and 20(*R*)-Rh2E2 on expression level of S phase specific Cdks/Cyclins. LLC-1 cells were treated with 20(*S*)/(*R*)-Rh2E2 (40 µM, 60 µM, 80 µM) for 24 h, respectively. Representative immunoblots and the protein quantification are shown; mean ± S.D., *n* = 3; *P < 0.05, **P < 0.01, one-way ANOVA analysis.

Metabolic regulation could influence cell proliferation and cell cycle progression^34^. Therefore, we investigated and compared the effect of 20(*S*)-Rh2E2 with 20(*R*)-Rh2E2 on the progression of cell cycle in LLC-1 cells. Results indicated that 20(*S*)-Rh2E2 was able to arrest the LLC-1 cells in S-phase (Fig. 5a). The percentage of cells in the S-phase was increased up to 41.30 ± 6.59% in LLC-1 cells treated with 80 μM of 20(*S*)-Rh2E2, which was significantly higher than that of the dimethyl sulfoxide (DMSO)-treated cells (17.97 ± 5.29%) (*P* < 0.01). These data suggested that 20(*S*)*/*(*R*)-Rh2E2 can effectively manipulate the proliferation of LLC-1 cells by arresting them in the S-phase of cell cycle. We then determined the expression profile of the molecular regulators responsible for S-phase cell cycle arrest upon Rh2E2 treatment, since it has been reported that inhibition of the cyclin-dependent kinase (Cdk)/Cyclin complex activity suppresses cell cycle progression^35^. In line with Fig. 5b and Supplementary Fig. S7, 20(*S*)- and 20(*R*)-Rh2E2 suppressed the expression of Cyclin D and Cdk4, with a concurrent decline in Cyclin A, while the expression of Cyclin E and Cdk2 was reduced. These findings are in agreement with our previous studies that 20(*R*)-Rh2E2 arrests LLC-1 cells in S-phase of cell cycle^13^.

### Expression of p27 in 20(*S*)-Rh2E2-treated LLC-1 cancer cells relies on inhibition of the S-phase kinase-associated protein 2 (Skp2) autoinduction loop

**Fig. 6:**
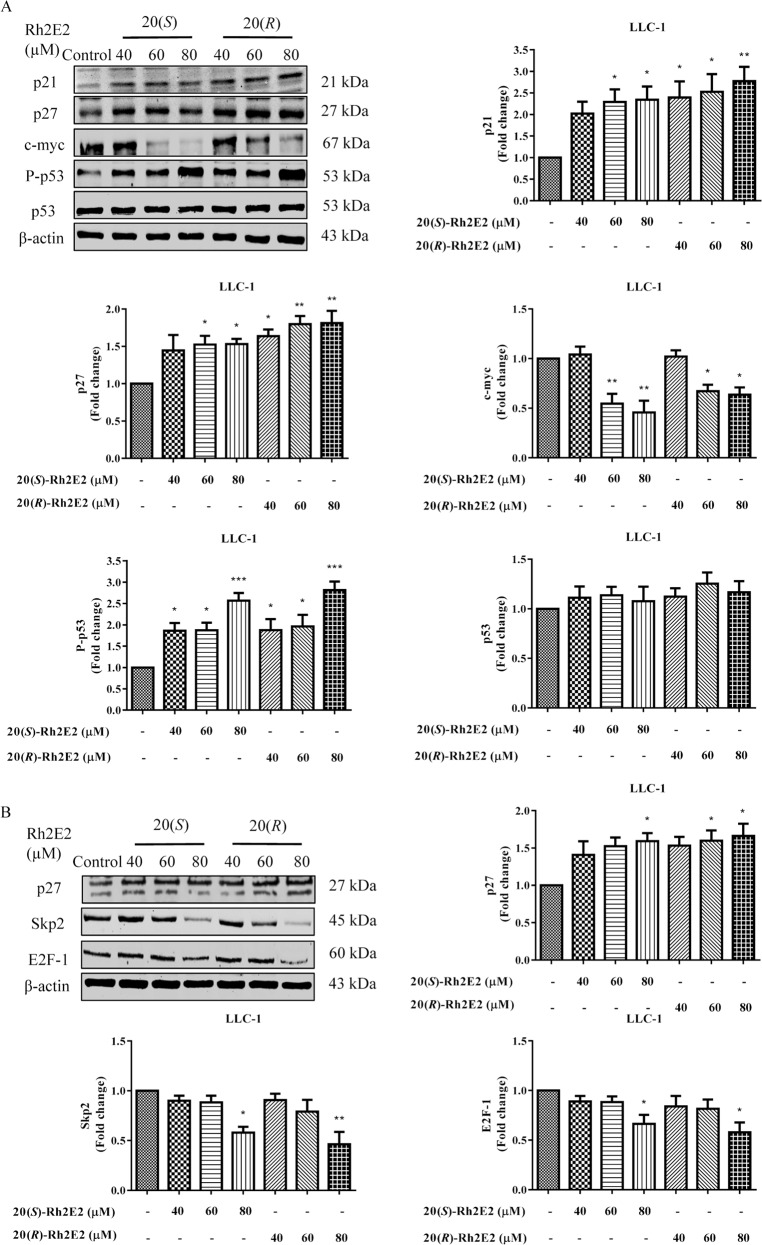
The role of Skp2 in 20(*S*)-Rh2E2-induced anti-cancer effect. **a** Effect of 20(*S*)-Rh2E2 and 20(*R*)-Rh2E2 on the expression of Cdk inhibitors during cell cycle progression of LLC-1 cells. **b** Effect of 20(*S*)-Rh2E2 and 20(*R*)-Rh2E2 on Skp2 signaling pathway in LLC-1 cells. LLC-1 cells were treated with 20(*S*)/(*R*)-Rh2E2 (40 µM, 60 µM, 80 µM) for 24 h, respectively. Representative immunoblots and the protein quantification are shown; mean ± S.D., *n* = 3; *P < 0.05, **P < 0.01, ***P < 0.001, one-way ANOVA analysis.

p21 is known to inhibit the activity of Cdk4/Cyclin D complex, while p27 can inhibit the activity of Cdk2/Cyclin E complex^36^. Previous studies have showed that the tumor suppressor proteins p21 and p27, as well as p53, could be able to suppress the AMPK-related metabolic pathway and glucose metabolism^37^. Therefore, 20(*S*)-Rh2E2 may downregulate cancer cell metabolism and arrest cell cycle progression *via* activation of these tumor suppressors. As expected, 20(*S*)- and 20(*R*)-Rh2E2 caused the accumulation of p21 and p27 in a dose-dependent manner with concomitant activation of p53. The expression of c-myc, which acts as a transcriptional factor for Cdks^38^, was also decreased (Fig. 6a and Supplementary Fig. S8). These findings suggested that 20(*S*)*/*(*R*)-Rh2E2 could enhance the expression of p21 and p27, which would bind and suppress the interaction between Cdks and Cyclins, leading to a loss of Cdks/Cyclins complex activity, and contributing to S-phase cell cycle arrest and cell cytotoxicity.

Skp2 plays a critical role in coordinating the G1/S transition and progression^39^. Ubiquitylation and degradation of p27 are important for Skp2-mediated entry to the cell cycle, and overexpression of Skp2 have been shown clearly associated with tumorigenesis^40^. Of note, 20(*R*)-Rh2E2 has been demonstrated to arrest cancer cells in S-phase *via* suppression of Skp2 autoinduction loop^13^. Here, we hypothesized that 20(*S*)-Rh2E2 may also interrupt cancer cell energy production and cell cycle progression *via* the Skp2 autoinduction loop. As shown in Fig. 6b and Supplementary Fig. S9, an increase in p27 was accompanied by a reduction of Skp2 and E2F-1 expression. These data suggest that both Rh2E2 compounds could arrest the cancer cells in S-phase *via* the same mechanisms of action.

### 20(*S*)-Rh2E2-mediated cell cytotoxicity requires the activation of AMPK-extracellular signal-regulated kinase (ERK) signaling

**Fig. 7:**
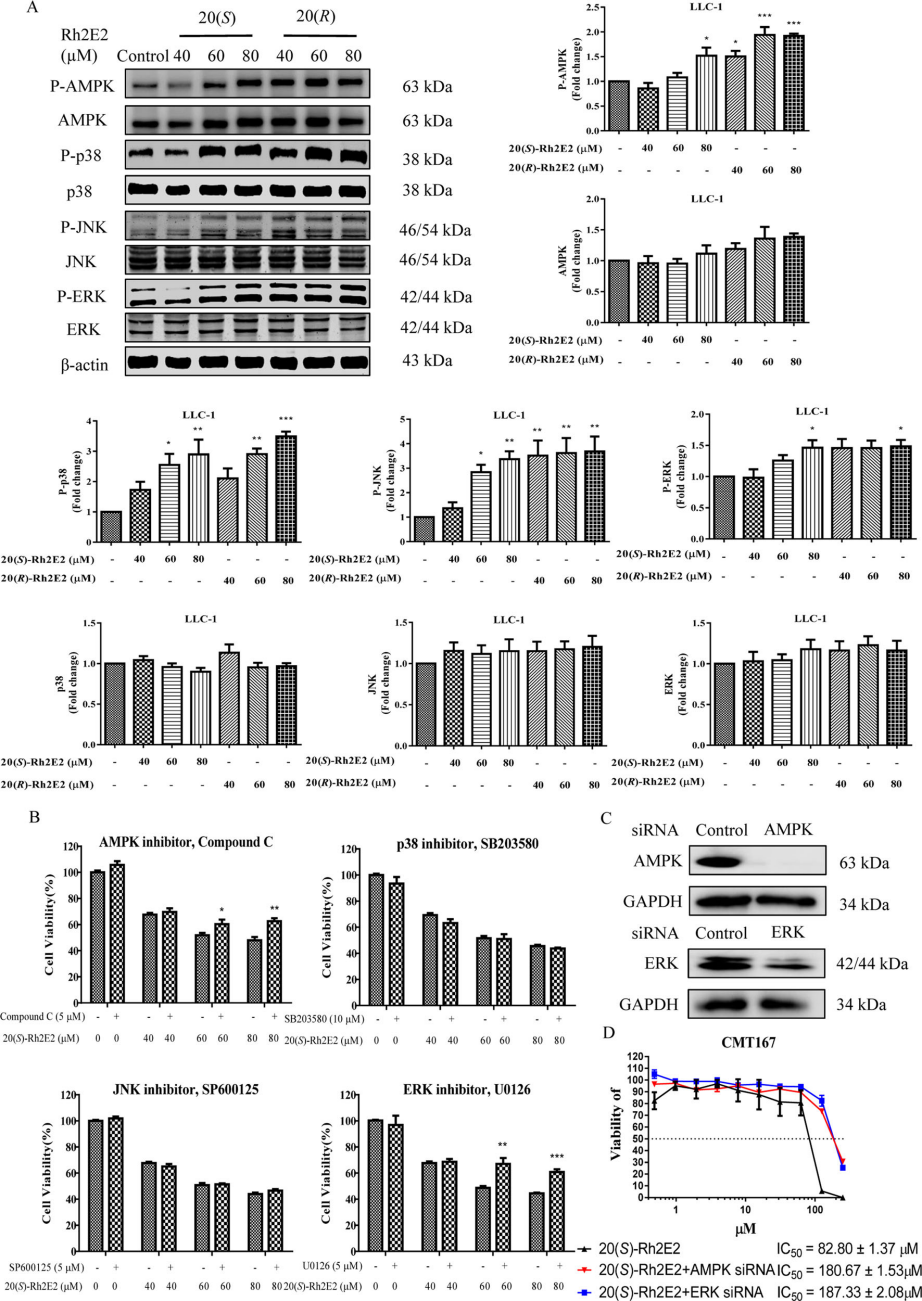
Effect of 20(*S*)-Rh2E2 on AMPK-MAPKs signaling pathway. **a** 20(*S*)-Rh2E2 and 20(*R*)-Rh2E2 activated AMPK and MAPK signaling *via* activation phosphorylation of p38, p-JNK, and p-ERK. LLC-1 cells were treated with 20(*S*)/(*R*)-Rh2E2 (40 µM, 60 µM, 80 µM) for 24 h, respectively. Representative immunoblots and the protein quantification are shown; mean ± S.D., *n* = 3; *P < 0.05, **P < 0.01, ***P < 0.001, one-way ANOVA analysis. **b** 20(*S*)-Rh2E2 induced cell cytotoxicity through activation of AMPK and MAPK signaling. LLC-1 cells were preincubated with or without specific inhibitors for 2 h, then treated with 20(*S*)-Rh2E2 together with or without specific inhibitors. After 72 h, the cell viability was measured by MTT assay. **c** siRNA knockdown of AMPK and ERK in CMT167 mouse lung cancer cells. **d** 20(*S*)-Rh2E2-mediated cell cytotoxicity was dependent on AMPK and ERK expression. Cytotoxic effect of 20(*S*)-Rh2E2 in CMT167 cells transfected with control nonspecific siRNA or AMPK- or ERK-targeted siRNA. siRNA transfected cells were treated with DMSO (control) or 20(*S*)-Rh2E2 at indicated drug concentrations for 48 h and then subjected to MTT assay. Mean ± S.D. are from three independent experiments (*P < 0.05, **P < 0.01, ***P < 0.001).

Mitogen-activated protein kinase (MAPK) signaling contributes to the maintenance of glucose homeostasis and peripheral tissue energy balance^41^. Among these, the ERK signaling pathway plays a key role in several steps of tumorigenesis^42^. c-Jun N-terminal kinase (JNK) is the final facilitator for ERK to stimulate cell proliferation^43^, whereas p38 is a tumor suppressor, which was quickly inactivated concomitant with robust tumor growth and metastatic behavior in the tumorigenic cells^44^. Our western blot results showed that the phosphorylation form of AMPK, p38, JNK, and ERK were markedly upregulated in a dose-dependent manner with the treatment of either 20(*S*)- or 20(*R*)-Rh2E2, indicating that both compounds activated AMPK as well as MAPKs signaling (Fig. 7a and Supplementary Figs. S10, S11). To further confirm the activation of these signaling pathways is necessary for 20(*S*)-Rh2E2-mediated cell cytotoxicity, LLC-1 cells were incubated with 20(*S*)-Rh2E2 in the presence of AMPK, p38, JNK, and ERK specific inhibitors. Cytotoxicity assay revealed that, compound C and U0126 exhibited a protective effect on 20(*S*)-Rh2E2-treated cells, and the viability of cells was increased to a certain extent (Fig. 7b). Concomitantly, we further performed the knockdown of AMPK and ERK in another mouse lung cancer cells, CMT167, because LLC-1 is not sensitive to siRNA transfection. Upon knockdown of AMPK and ERK in CMT167 cells (Fig. 7c and Supplementary Fig. S12), the cell viability could be partially recovered from cell death induced by 20(*S*)-Rh2E2 (Fig. 7d), suggesting that AMPK and ERK activation were necessary for 20(*S*)-Rh2E2-induced cell cytotoxicity. Activation of AMPK signaling would also have chance to induce autophagy^45–47^. Accordingly, we examined the autophagy effect of 20(*S*)*/*(*R*)-Rh2E2 in HeLa cells by western blot and immunocytochemical staining. As shown in Supplementary Fig. S13, HeLa cells treated with 20(*S*)- or 20(*R*)-Rh2E2 demonstrated no autophagy signal, as presented by the absent of autophagic marker LC3-II conversion, and the absent of red endogenous LC3 puncta formation (red TRITC signal).

## Discussion

Previous reports showed that 20(*S*)-Rh2 could reduce the transcriptional activity of E2F by targeting cyclin D1 and CdK4/6, leading to G1/S cell cycle arrest and apoptosis in liver cancer and breast cancer cells^48^. Besides, 20(*S*)-Rh2 could also promote JNK and ERK1/2 activation and induce apoptosis in human lung adenocarcinoma A549 cells^49^. However, 20(*S*)-Rh2 has never been reported to regulate the cancer cells energy metabolism so far. Intriguingly, 20(*S*)-Rh2E2 not only activated MAPKs signaling pathways, but also activated the energy sensor AMPK signaling cascade, suggesting that 20(*S*)-Rh2E2 might be equipped with new anti-tumor property in comparison with its parental compound 20(*S*)-Rh2. Notably, 20(*S*)-Rh2 has been showed to exhibit cytotoxicity toward normal cells^50^. Here, we revealed that 20(*S*)-Rh2E2 specifically suppressed cancer cell energy metabolism and reduced acetyl CoA and ATP production in LLC-1 cells, but not normal cells. In contrast, CCD19Lu cells treated with 20(*S*)-Rh2E2 showed an increase of ATP production, and animal administrated with high dose of 20(*S*)-Rh2E2 showed no animal dead or significant reduction in body weight and vital organs, suggesting the beneficial effect of non-toxic property and tumor specificity of 20(*S*)-Rh2E2 compared with 20(*S*)-Rh2. Nevertheless, the therapeutic efficacy of 20(*S*)-Rh2 and 20(*S*)-Rh2E2 has not been compared in present study, since our previous work already demonstrated that IP injection and oral administration of 20(*S*)-Rh2 at the concentration of 10 or 80 mg/kg/day exhibited no anti-tumor effect^13^. Here, we successfully demonstrated the in vivo anti-tumor and anti-metastatic effect of 20(*S*)-Rh2E2 in comparison with 20(*R*)-Rh2E2.

Administration of 20(*S*)-Rh2E2 to LLC-1 xenograft mouse model via the IP route effectively suppressed tumor growth and metastasis, showing a large range of safety window. In addition, the glycolytic enzyme α-enolase and the metabolic enzymes involve in fatty acid β-oxidation were downregulated by 20(*S*)-Rh2E2, leading to reduced production of ATP and acetyl CoA. In consistent to these findings, a few studies found that tumor cells with hampered fatty acid oxidation are more sensitive to metabolic stress^51^. Intriguingly, pharmacological inhibition of fatty acid oxidation profoundly decreased energy metabolism can block tumor growth^52^. In this study, 20(*S*)-Rh2E2 most probably reduces cancer cell metabolism, thereby, arresting cancer cell growth in the S-phase via Skp2 signaling as observed. Skp2 controls cell proliferation, especially promoting S-phase entry by the degradation of p27. In fact, our western-blot analysis demonstrated an upregulation of p27 expression upon 20(*S*)-Rh2E2 treatment. Some studies showed that Skp2 deficiency impairs Akt activation, glucose transporter 1 expression, and glucose uptake and glycolysis, suppressing cancer progression in various tumor models^53^. It is worth noting that a newly identified Skp2 inhibitor, with the use of other chemotherapeutic agents, has been shown to suppress Akt-mediated glycolysis and trigger p53-independent cellular senescence in multiple animal models to reduce cancer cell growth^54^. Therefore, 20(*S*)-Rh2E2 is a potent anti-cancer agent by suppressing Skp2.

On the other hand, α-enolase also acts as a plasminogen receptor and thus mediates activation of plasmin and extracellular matrix degradation^55^. In cancer cells, the expression of α-enolase is upregulated at the cell surface, where it promotes cancer invasion, and is subjected to a specific array of post-translational modifications^56,57^. The overexpression of α-enolase and its post-translational modifications could be of diagnostic and prognostic value in cancer^56^. In addition, our results revealed that 20(*S*)-Rh2E2 suppressed the basal glycolysis and glycolytic capacity of LLC-1 cells, which implies the decrease of lactic acid production and release. Since, the uplifted extracellular acidity favors tumor metastatic dissemination and lactate itself directly contributes to the migration of cells and cell clusters^58^, the reduced invasiveness of 20(*S*)-Rh2E2-treated LLC-1 cells is likely to be a result of inhibited glycolysis by the suppression of α-enolase.

Finally, we discovered the important role of MAPKs in controlling cellular responses to the cancerous microenvironment through the regulation of gene expression, cell growth, and apoptosis, which bestow them the priority for research related to cancer therapy. In our study, the suppression of cell viability was significantly abrogated in 20(*S*)-Rh2E2-treated cells in the presence of AMPK and ERK inhibitor, indicating that the activation of AMPK and ERK is implicated in 20(*S*)-Rh2E2-induced cytotoxicity in LLC-1 cells. Most importantly, no adverse side effects have been observed in LLC-1 xenograft model upon 20(*S*)-Rh2E2 treatment at the effective dosages which is higher than that of other chemotherapeutic agents such as the first-line anti-cancer agent paclitaxel. Moreover, the AMPK-ERK signaling is a potential molecular target for drug development, and inhibitors of this molecular signaling could be one of the next groups of compound for the effective treatment of tumorigenesis^59^.

Taken together, 20(*S*)-Rh2E2 enhances tumor shrinkage and metastasis for lung cancer by specifically suppressing cancer cell metabolism via the inhibition of metabolic enzymes in mitochondrion. It represses the proliferation of LLC-1 cells by inducing S-phase cell cycle arrest, which provides molecular details for in-depth study of tumor metabolism. 20(*S*)- and 20(*R*)-Rh2E2 effectively suppress cancer growth with no major adverse effects. These findings are particularly important, since apart from the classical case of thalidomide, of which one enantiomer demonstrate pharmacological efficacy while another with catastrophic side effects, other cases are not uncommon. For example, bupivacaine typically used in the racemate form as local anesthetic, the (*S*)-enantiomer appeared to have a higher cardiotoxicity than the (*R*)-enantiomer^60^. Accordingly, the guidelines issued by The United States Food and Drug Administration request that the pharmacological effect of individual stereoisomers in any new racemic mixture should be characterized at the early phase of drug development process^53,61^. In addition, the simultaneous existence of 20(*R/S*)-Rh2E2 enantiomers during their synthesis process from their mixed precursors 20(*R/S*)-Rh2 is unavoidable and the complex chiral separation of 20(*R/S*)-Rh2 is highly expensive. Therefore, our findings support the practicality of using 20(*R/S*)-Rh2E2 as effective anti-cancer drugs.

## Materials and methods

### Preparation of 20(*S*)-Rh2E2

A mixture of Oxone® mono-persulfate compound (4942 mg) and NaHCO_3_ (2104 mg) was added slowly to a solution of 20(*S*)-Rh2 (1000 mg) in 600 mL of a 1:1 mixture of acetonitrile-Na_2_(EDTA) (4 × 10^−4^ M in water). Then, shi epoxidation diketal catalyst (Ketone, 1245 mg) in 150 mL of acetronitrile was added dropwise during a period of 10 min. The reaction mixture was allowed to stand overnight at room temperature and magnetically stirred. After filtration and removal of acetronitrile in vacuum, the reaction solution was directly loaded to an ODS column and eluted from 50 to 90% methanol to afford 20(*S*)-Rh2 20,24-epoxides (700 mg) in an equivalent mixture of 24-epimers, i.e., 3-O-*β*-D-glucopyranosyl 20*S*,24*S*-epoxydammarane-3*β*,12*β-*triol and 3-O-*β*-D-glucopyranosyl 20*S*,24*R*-epoxydammarane-3*β*,12*β-*triol (20(*S*)-Rh2E2) as illustrated in Fig. 1a.

Structural identification of 3-O-*β*-D-glucopyranosyl 20*S*,24*S*-epoxydammarane-3*β*,12*β-*triol, and its 24*R* epimer: White amorphous powder. High Resolution-ESI-MS (Positive ion mode): *m/z* 639.4480 [M+H]^+^ (calculated for C_36_H_63_O_9_: 639.4467). ^1^H-NMR (600 MHz, C_5_D_5_N) *δ*: δ 4.98 (2H, d, *J* = 8.4 Hz, H-1′), 4.64 (2H, d, *J* = 11.6 Hz, H-6′a), 4.44 (2H, dd, *J* = 11.6 4.2 Hz, H-6′b), 4.31 (2H, m, H-3′), 4.24 (2H, m, H-4′), 4.22 (1H, t, *J* = 6.0 Hz, H-24, 24*S*-epimer), 4.09 (2H, m, H-2′), 4.06 (2H, m, H-5′), 3.99 (1H, t, *J* = 6.6 Hz, H-24, 24*R*-epimer), 3.80 (2H, m, H-12), 3.41 (2H, dd, *J* = 11.4 4.2 Hz, H-3), 1.51, 1.50 (3H each, s, H-26), 1.36 (3H, s, H-27), 1.36, 1.35 (3H each, s, H-28), 1.33 (3H, s, H-21), 1.31 (6H, s, H-27, H-21), 1.04, 0.10 (3H each, s, H-30) 1.04, 1.00 (3H each, s, H-29), 0.96, 0.94 (3H each, s, H-19), 0.86, 0.79 (3H each, s, H-18).

^13^C-NMR (150 MHz, C_5_D_5_N) *δ*: δ 39.83 and 39.78 (C-1), 27.30 and 27.26 (C-2), 89.4 and 89.3 (C-3), 40.3 and 40.2 (C-4), 57.03 and 57.00 (C-5), 19.1 and 19.0 (C-6), 35.72 and 35.67 (C-7), 40.6 and 40.5 (C-8), 51.3 and 51.1 (C-9), 37.6 and 37.5 (C-10), 33.0 and 32.7 (C-11), 71.7 and 71.4 (C-12), 50.03 and 49.97 (C-13), 57.03 and 57.00 (C-14), 29.3 and 29.2 (C-15), 33.2 and 33.1 (C-16), 50.2 and 48.9 (C-17), 18.9 and 18.6 (C-18), 17.14 and 17.09 (C-19), 87.7 and 87.3 (C-20), 29.6 (C-21, 24*S*-epimer), 27.50 (C-21, 24*R*-epimer), 26.4 and 26.0 (C-22), 32.2 and 33.4 (C-23), 89.0 (C-24, 24*S*-epimer), 86.1 (C-24, 24*R*-epimer), 71.0 and 70.7 (C-25), 27.53 (C-26, 24*R*-epimer), 27.1 (C-26, 24*S*-epimer), 28.0 (C-27, 24*R*-epimer) 27.7 (C-27, 24*S*-epimer), 28.69 and 28.66 (C-28), 17.33 and 17.29 (C-29), 16.2 and 16.1 (C-30), 107.5 (C-1′), 76.3 (C-2′), 79.3 (C-3′), 72.4 (C-4′), 78.9 (C-5′), 63.59 and 63.57 (C-6′).

### Cell culture

A549, H1299, Hela, MCF7, HepG2, LLC-1, CCD19Lu, LO2, and CMT167 cell lines were purchased from American Type Culture Collection (Rockville, MD, USA). These cell lines were authenticated by ATCC. All media were supplemented with 10% foetal bovine serum, 50 U/ml penicillin, and 50 μg/ml streptomycin (Invitrogen, Paisley, Scotland, UK). Cells were cultured at 37 °C in a 5% CO_2_-humidified incubator.

### Chemicals, antibodies, and small interfering RNAs

All reagents and siRNAs were purchased from Santa Cruz Biotechnology, unless otherwise stated. The following reagents from other suppliers were used: phospho-p53 kinase (Ser^15^) rabbit mAb (CST, 9284, USA), p53 kinase rabbit mAb (CST, 9282, USA), phospho-p38 kinase (Thr^180^/Tyr^182^) rabbit mAb (CST, 4511, USA), p38 kinase rabbit mAb (CST, 8690, USA), phospho-JNK kinase (Thr^183^/Tyr^185^) rabbit mAb (CST, 4668, USA), JNK kinase rabbit mAb (CST, 9252, USA), phospho-ERK kinase (Thr^202^/Tyr^204^) rabbit mAb (CST, 4370, USA), ERK kinase rabbit mAb (CST, 4695, USA), α-enolase kinase rabbit mAb (CST, 3810, USA), stathmin kinase rabbit mAb (CST, 3352, USA), phospho-AMP-activated protein kinase (AMPK; Thr^172^) rabbit mAb (CST, 2531, USA), AMPK rabbit mAb (CST, 2532, USA), anti-β-of actin mouse monoclonal IgG1 (Santa Cruz, sc-47778, USA), rabbit anti-mouse IgG (H +L) secondary antibody TRITC (Invitrogen, PA1-28565, USA), IRDye 800CW goat anti-mouse IgG (H + L) secondary antibody (Li-COR, 926-32210, USA), IRDye 800CW goat anti-rabbit IgG (H + L) secondary antibody (Li-COR, 926–32211, USA), Compound C (Calbiochem, 171260, USA), SB203580 (CST, 5633, USA), SP600125 (CST, 8177, USA), U0126 (CST, 9903, USA), antibody against LC3-II (CST, 2775, USA).

### Cytotoxicity assay

Cell viability and the inhibitory concentration (IC) were determined by 3-(4,5-dimethylthiazol-2-yl)-2, 5-diphenyltetrazolium bromide (MTT) assay. 20(*S*)- and 20(*R*)-Rh2E2 were dissolved in DMSO at a final concentration of 50 mmol/l and stored at −40 °C before use. Briefly, cells were seeded in 96-well plates and then exposed to test compounds at different concentrations or DMSO as a control for 72 h. Subsequently, MTT (10 μl) was added to each well for 4 h followed by the addition of 100-μL solubilization buffer (10% sodium dodecyl sulfate in 0.01 mol/L HCl) and overnight incubation. Color intensity was measured at 570 nm using a microplate reader. The percentage of cell viability was calculated by the formula: Cell viability (%) = (*A*_treated_ – *A*_background_)/(*A*_control_ – *A*_background_) × 100.

### LLC-1 Xenograft model and In vivo metastasis assay

Male C57BL/6 mice at the age of 6–8 weeks were obtained from The Chinese University of Hong Kong. All experiments were carried out in accordance with the “Institutional Animal Care and User Committee guidelines” of the Macau University of Science and Technology. Mice were subcutaneously injected with LLC-1, randomly divided into five groups. 20(*S*)- and 20(*R*)-Rh2E2 were dissolved in polyethylene glycol 400 (PEG400): ethanol: ddH_2_O = 6:1:3, and given by intraperitoneal injection at doses of 10 and 20 mg/kg continuously for 21 days. Body weight and tumor volumes (length × width^2^ × 1/2) were measured every day. To determine the lethal dose of 20(*S*)-Rh2E2, the mice were orally administered with 320 mg/kg 20(*S*)-Rh2E2 for 7 consecutive days (*n* = 4). The H&E-stained lung sections, taken at 50 μm intervals, were examined by microscope for metastatic lesions. Samples were imaged by Leica DFC310 FX camera and lung areas were calculated by Leica Application Suit V4.4 software. The percentage of metastatic lung area was calculated as metastatic burden area/lung area.

### Immunohistochemistry (IHC)

The dissected tumor tissues were fixed and then processed into paraffin blocks for sectioning at 5 µm. Mounted tissue sections were deparaffinized in xylene, and subsequently rehydrated in graded ethanol and ddH_2_O. For antigen retrieval, citrate buffer (Sigma) was used for 20 min at 99 °C. Then, 3% of hydrogen peroxide was applied for 10 min at room temperature to block the endogenous peroxidase activity. Anti-α-enolase or anti-stathmin antibody was incubated as primary antibody overnight at 4 °C. The secondary antibody, SuperPicture^TM^ HRP Polymer conjugate (ZYMED Lab., Invitrogen, Carlsbad, CA), was added for 1 h. After washing, slides were incubated in 3,3′-diaminobenzidine (DAB) substrate solution until the desired stain intensity was developed. The slides were then counterstained with hematoxylin, dehydrated and mounted. Immunostaining images were captured by Leica DM2500 microscope.

### Protein preparation and western blot analysis

After drug treatment, adherent and floating cells were lysed with radioimmunoprecipitation assay buffer (CST, 9806, USA). Protein concentrations were determined by using the Bio-Rad protein assay (Bio-Rad Laboratories, Inc., Hercules, CA, USA). Cell lysates were subjected to electrophoresis on sodium dodecyl sulfate polyacrylamide gel electrophoresis (SDS-PAGE). After electrophoresis, samples were transferred to a polyvinylidene (PVDF) membrane or nitrocellulose (NC) membrane, and then incubated in blocking buffer at room temperature for 1 h. Immunoblotting was performed by treating the membrane with primary antibodies at 4 °C overnight followed by secondary antibodies. Proteins detection was performed using Amersham Imager 600 (GE Healthcare Life Sciences) chemiluminescence (Invitrogen, USA) or Odyssey CLxImager (Li-COR, Lincoln, NE, USA). Band intensities were quantified by using the software ImageJ (NIH, Bethesda, MD, USA) or Odyssey v3.0 software (Li-COR, USA).

### Wound healing migration assay

The wound-healing assay was used to assess the in vitro migration ability of cancer cells. Briefly, six-well plates were seeded with H1299 cells. After 24 h, when cells grew to 90% confluency, cell monolayers were scratched with a sterile micropipette tip. In the next step, wounded monolayers were washed with phosphate-buffered solution (PBS) to remove cell debris and maintained with serum-free medium at 37 °C and 5% CO_2_. Wound closure was monitored by visual examination at the time points (0 and 24 h) using an inverted bright field microscope.

### Transepithelial electrical resistance (TEER) treatment

Before measurement, electrodes were equilibrated and sterilized according to the manufacturer’s recommendations. In brief, the prechilled pipet tips were used and the pipet were held perpendicular to the well, 40 μl of prepared Matrigel was seeded to the center of filter insert of the pre-colded 24-well plate, and incubated in 37 °C incubator until solidified. To eliminate the influence of temperature, measurements were performed quickly after taking the culture plates out of the incubator. Two hundred microliters of serum-free culture medium containing H1299 cells at a density of 1 × 10^5^ cells/well was added in the upper compartment of the cell culture system. TEER24 base plate wells was filled with 1 ml of media containing serum. The ohmic resistance of a blank (culture insert without cells) was measured in parallel. To obtain the sample resistance, the blank value was subtracted from the total resistance of the sample. The final unit area resistance (Ω*cm^2^) was calculated by multiplying the sample resistance by the effective area of the membrane (0.33 cm^2^ for 24-well Millicell inserts).

### Seahorse XF metabolic stress assay

LLC-1 and CCD19Lu cells were plated in XFp Cells Culture Miniplates (Agilent, Santa Clara, CA, USA). After 48 h, glycolysis or mitochondrial respiration was determined with XFp Cells Glycolysis Stress Test Kit or XFp Cells Mito Stress Test Kit on Seahorse Bioscience XFp extracellular flux analyzer (Agilent, Santa Clara, CA, USA). Glucose (100 mM), oligomycin (10 μM) and 2-deoxyglucose (2-DG, 500 mM) were serially injected to measure the extracellular acidification rate (ECAR). The mitochondrial oxygen consumption rate (OCR) was measured by serial injection of oligomycin (10 μM), carbonyl cyanide 4-(trifluoromethoxy) phenylhydrazone (FCCP) (5 μM), 5 μM mix of rotenone (complex I inhibitor) and antimycin A (complex III inhibitor). Data analysis was performed with Seahorse XFp Analyzer Software (Agilent, Santa Clara, CA, USA).

### Liquid chromatography-mass spectrometry/mass spectrometry (LC-MS/MS) measurement of ATP metabolites

The treated cells were harvested in 12 ml of ice-cold PBS. The cell pellet was then treated with 150 μl of 15% trichloroacetic acid containing 7.5 μl of 20.0-μM [^13^C, ^15^N] ATP as internal standard and placed on ice for 10 min. After centrifugation at 12,100 × *g* for 15 min, the acidic supernatant was separated and neutralized twice with 80 μl mixture of trioctylamine and 1,1,2-trichlorotrifluoroethane (a volume ratio of 45–55), the samples were then ready for LC-MS/MS analysis. Data acquisition was performed with the Xcalibur software version 2.0.7, and data processing was carried out using the Thermo LCquan 2.5.6 data analysis program. The chromatographic separation was performed using Xterra-MS C18 column (150 mm × 2.1 mm i.d., 3.5 μm, Waters, Milford, MA). The two eluents were as follows: (A) 5mM hexylamine (HA)−0.5% diethanolamine (DEA) in water, pondus hydrogenii (pH) 10 was adjusted with acetic acid; and (B) 50% acetonitrile in water. The mobile phase consisted of linear gradients of A and B: 0–15 min, 100-80% A (v/v); 15–35 min, 80-70% A; 35–45 min, 70-45% A; 45-46 min, 45-0% A; 46–50 min, 0-0% A; and 51–70 min, 100-100% A. The liquid flow rate was set at 0.3 ml/min, and the column temperature was maintained at 35 °C.

### Acetyl-CoA assay

The Acetyl-CoA amount was determined by Acetyl-CoA Fluorometric Assay Kit (Biovision, K317-100, USA) following the manufacturer’s instruction. LLC-1 cells were treated with 80 µM 20(*S*)-Rh2E2 and 80 µM 20(*R*)-Rh2E2 for 24 h. First of all, 2 × 10^6^ Rh2E2-treated cells were homogenized and deproteinized on ice. The cell lysates were centrifuged at 10,000 × *g* for 10 min to remove insoluble material. The supernatant was replenished to a final volume of 50 µL with Acetyl-CoA assay reagent, mixed and incubated the reaction in 96 well plate for 10 min at 37 °C. After incubation, the absorbance of fluorescence intensity (λex = 535/λem = 587 nm) was detected by a plate reader and apply the sample readings to the Standard Curve to get the Acetyl-CoA amount in the sample wells. The calculation formula of Acetyl-CoA concentrations is as follow:

Concentration = *A*_y_/*S*_v_

*A*_y_ = amount of Acetyl-CoA (pmol) in sample from Standard Curve.

*S*_v_ = sample volume (μl) added to the reaction wells.

### α-KG assay

LLC-1 cancer cells treated with or without 80 µM 20(*R*)- or 20(*S*)-Rh2E2 were harvested for determination of α-KG by α-KG Assay Kit (Biovision, K677-100, USA) following manufacturer’s instruction. Firstly, 2 × 10^6^ LLC-1 cells were homogenized and deproteinized with 10 kDa molecular weight cut-off (MWCO) spin filter. After centrifugation, the supernatant was mixed with α-KG assay reagent and incubated in 96-well plate for 30 min at 37 °C. The mixture absorbance at 570 nm was further detected by the TECAN plate reader and the amount of α-KG was calculated based on the Standard Curve. The calculation formula of α-KG concentrations is as follow:

Concentration = *A*_y_/*S*_v_

*A*_y_ = amount of α-KG in sample from Standard Curve.

*S*_v_ = sample volume added to the reaction wells.

### Cell cycle analysis

The cells were harvested and washed with ice-cold PBS, and then suspended and permeabilized with 70% ethanol for 2 h at 4 °C. For detecting deoxyribonucleic acid (DNA) content and cell cycle, cells were incubated with the freshly prepared propidium iodide (PI) staining buffer for 30 min at room temperature in dark. All experiments were performed three times, respectively. The population of cells were quantitatively determined by flow cytometer (BD FACSAria III, San Jose, CA, USA).

### L-Lactate assay

The lactate concentration was measured using the colorimetric L-Lactate Assay Kit (Abcam, ab65331, USA) according to the manufacturer’s instruction. Briefly, the concentration of lactate in culture medium or cell lysates was detected by spectrophotometry at 450 nm using a standard curve generated with a known concentration of lactate solution. For the cellular lactate, LLC-1 and H1299 cells were sonicated with PBS and concentration of L-Lactate in the test samples was calculated as:

Lactate concentration = (La/Sv) * D

La = amount of lactic acid in the sample well calculated from standard curve (nmol).

Sv = volume of sample added into the well (μL).

D = sample dilution factor.

### Real-Time quantitative PCR

Gene expression was analyzed by real-time quantitative PCR with ViiA^TM^ 7 Real-Time PCR System (Applied Biosystems, Waltham, MA, USA) using PowerUp^TM^ SYBR^TM^ Green Mastermix (ThermoFisher Scientific, San Jose, CA, USA). The cDNA was prepared by using the Transcriptor Universal cDNA Master Kit (Roche, Basel, Switzerland). Primers sequence (see Supplementary Tables) synthesized by Tech Dragon Ltd (Hong Kong, China) were designed by employing the ThermoFisher Scientific’s online OligoPerfect^TM^ Designer software and further verified with NCBI’s Primer-BLAST software.

### Endogenous autophagy detection

The detection of endogenous LC3 was conducted using immunofluorescence staining method as described below. In brief, HeLa cancer cells on cover slips were fixed with 4% paraformaldehyde (Sigma, 158127-3KG, USA) for 20 min at room temperature and then rinsed with PBS. Coverslips were immersed in methanol at room temperature for 2 min. After washing with PBS, the cells were then incubated with anti-LC3 (1:200) in TBST (100 mM Tris HCl, pH 7.5, 150 mM NaCl, 0.05% Tween 20 and 5% BSA) overnight at 4 °C. After washing with PBS, the cells were incubated with anti-mouse secondary antibody (TRITC) (1:200) in TBST containing 5% BSA at 37 °C for 1 h in the dark. The coverslips were then mounted with FluorSave™ mounting media (Calbiochem, 345789, USA). Samples were imaged by widefield epifluorescence microscopy using Photometrics CoolSNAP HQ2 CCD camera on the Olympus IX71-Applied Precision DeltaVision restoration microscope (Applied Precision Inc, USA). All fluorescence images were deconvolved using DeltaVision algorithms (Applied Precision, Inc). The percentage of cells with autophagic induction was calculated by the number of the cells with increased formation of punctate LC3 fluorescence dots (≥10 dots/cell) over the total number of immunofluorescence-positive cells in the same field. A minimum of 1000 cells from randomly selected fields were scored.

## Supplementary information

Supplementary Figure S1

Supplementary Figure S2

Supplementary Figure S3

Supplementary Figure S4

Supplementary Figure S5

Supplementary Figure S6

Supplementary Figure S7

Supplementary Figure S8

Supplementary Figure S9

Supplementary Figure S13

Supplementary Figure S10

Supplementary Figure S11

Supplementary Figure S12

Supplementary Table S1

Supplementary Table S2

Supplementary Table S3
