# Supplementary figures and images for: Novel ginsenoside derivative 20(S)-Rh2E2 suppresses tumor growth and metastasis in vivo and in vitro via intervention of cancer cell energy metabolism
Source: Cell Death Dis. 2020 Aug 14;11(8):621. doi: 10.1038/s41419-020-02881-4 (PMC7427995; doi:10.1038/s41419-020-02881-4)

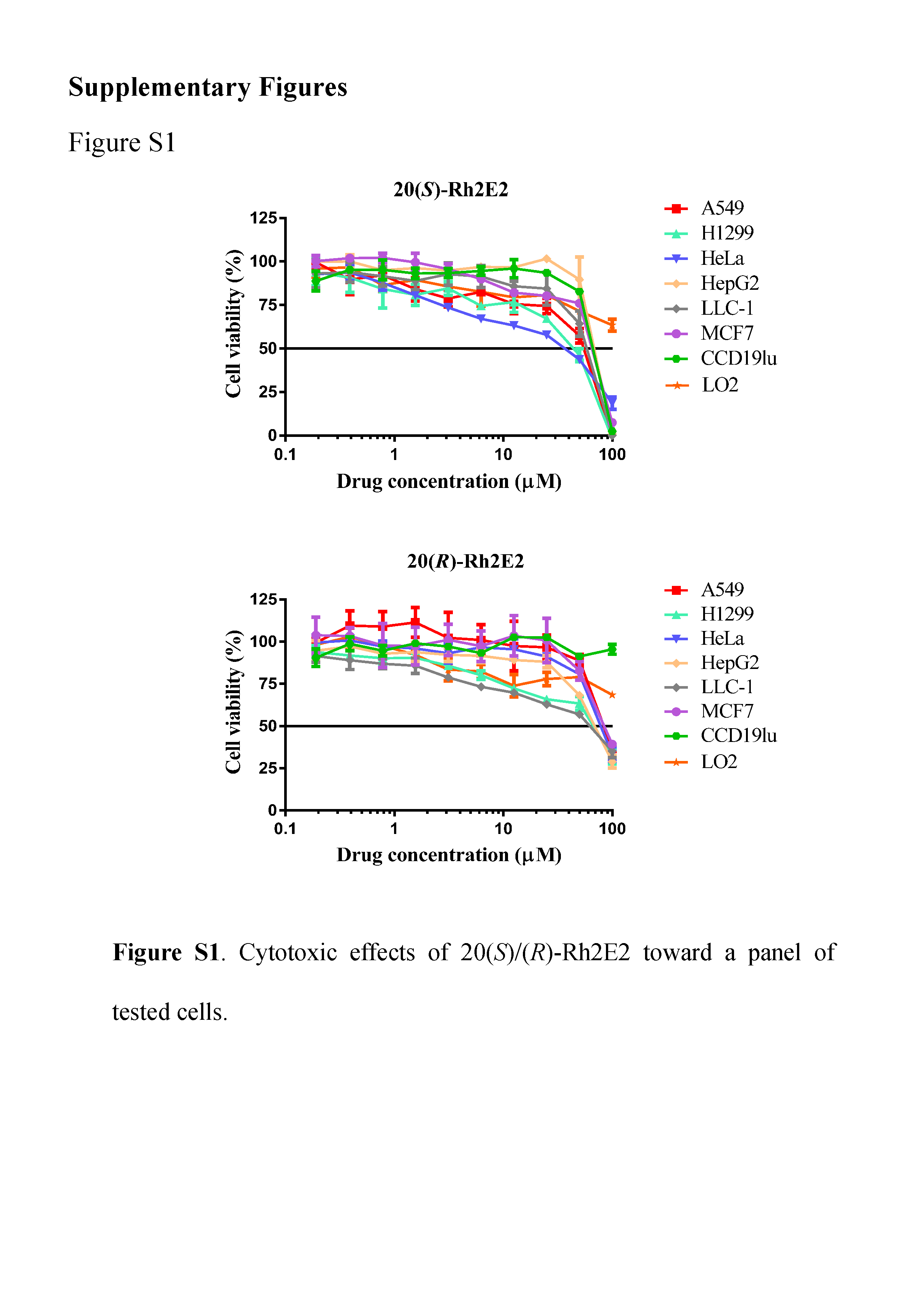

Supplement: Supplementary file 1 — Supplementary Figure S1 [file 41419_2020_2881_MOESM1_ESM.tif]

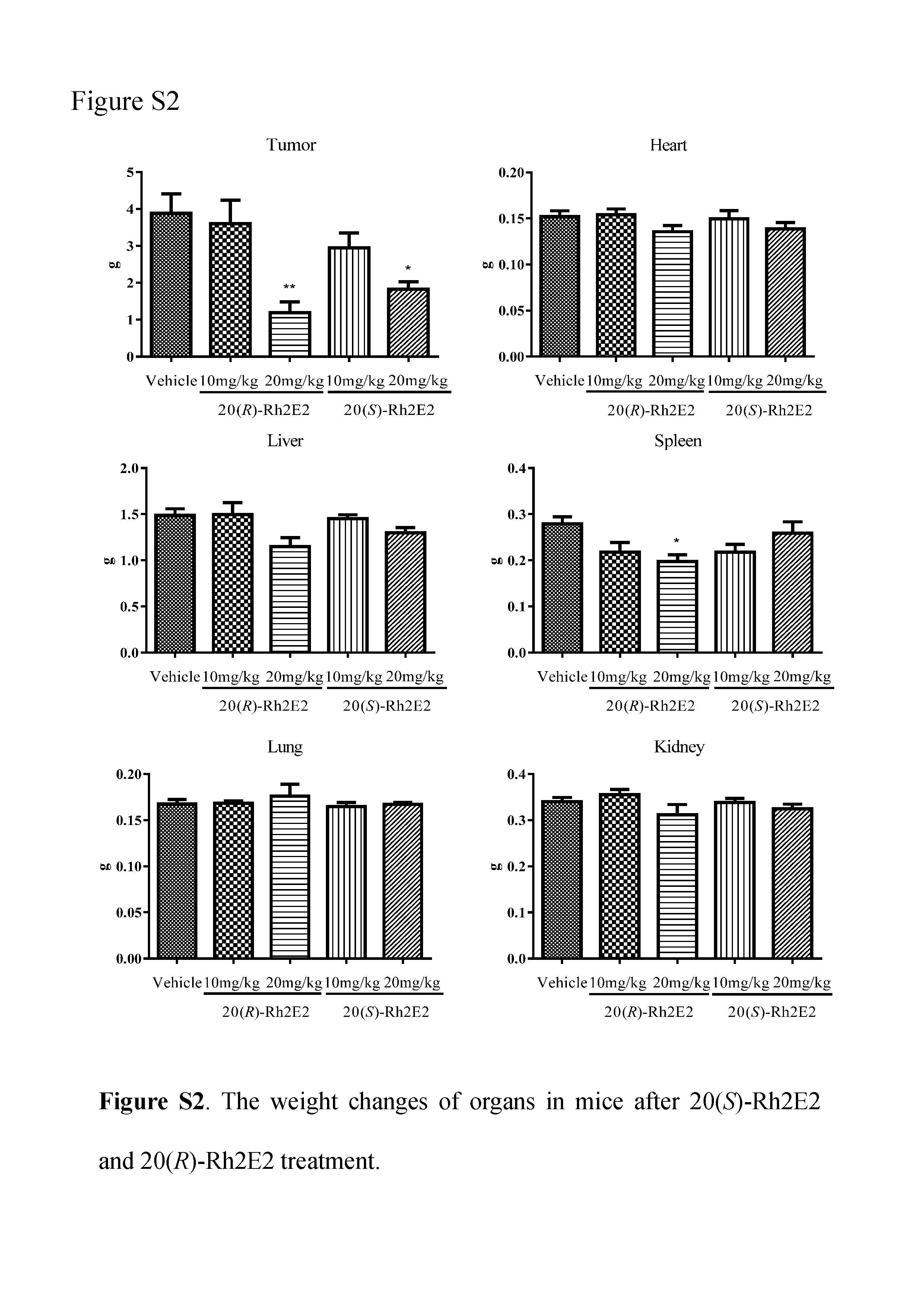

Supplement: Supplementary file 2 — Supplementary Figure S2 [file 41419_2020_2881_MOESM2_ESM.tif]

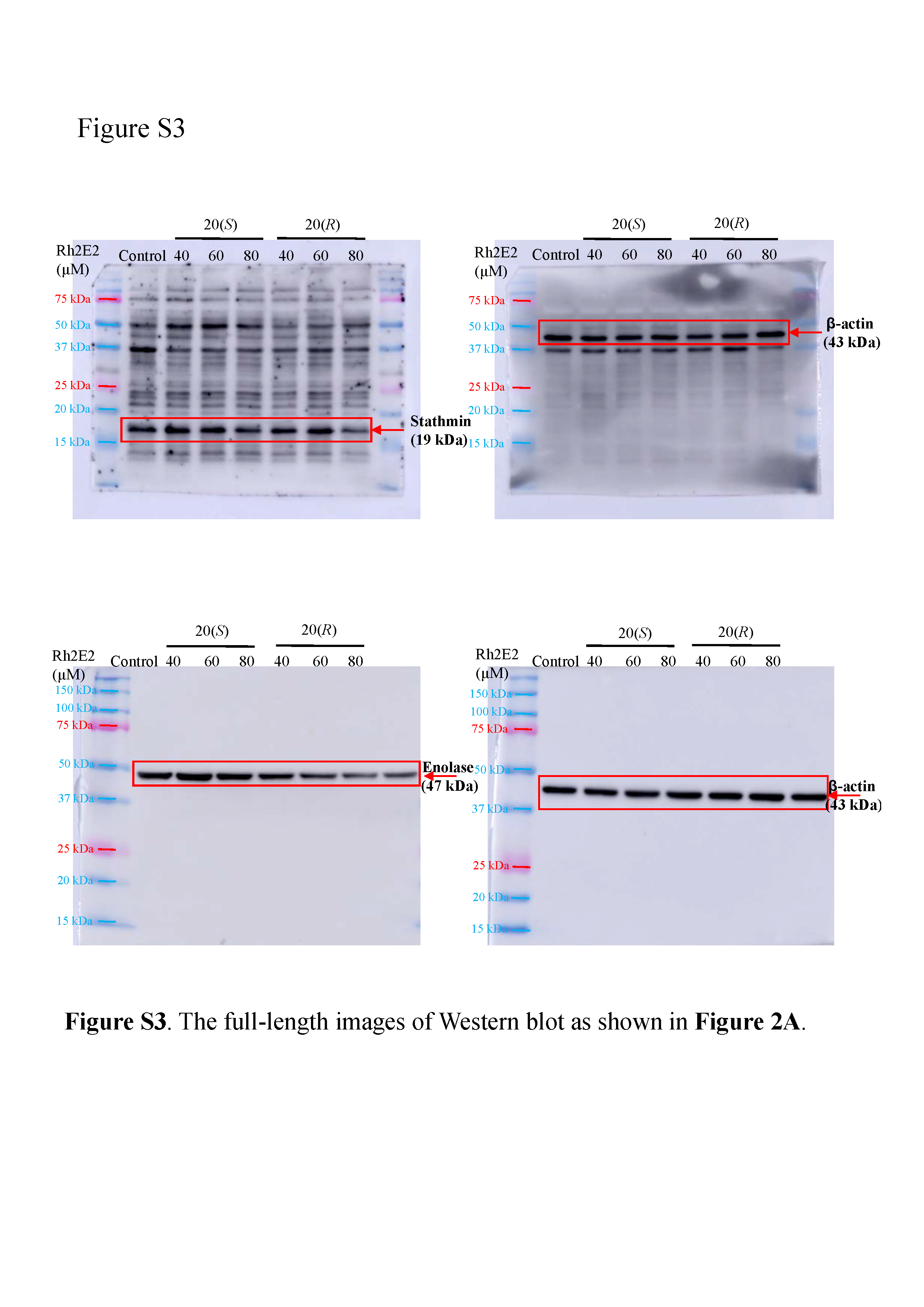

Supplement: Supplementary file 3 — Supplementary Figure S3 [file 41419_2020_2881_MOESM3_ESM.tif]

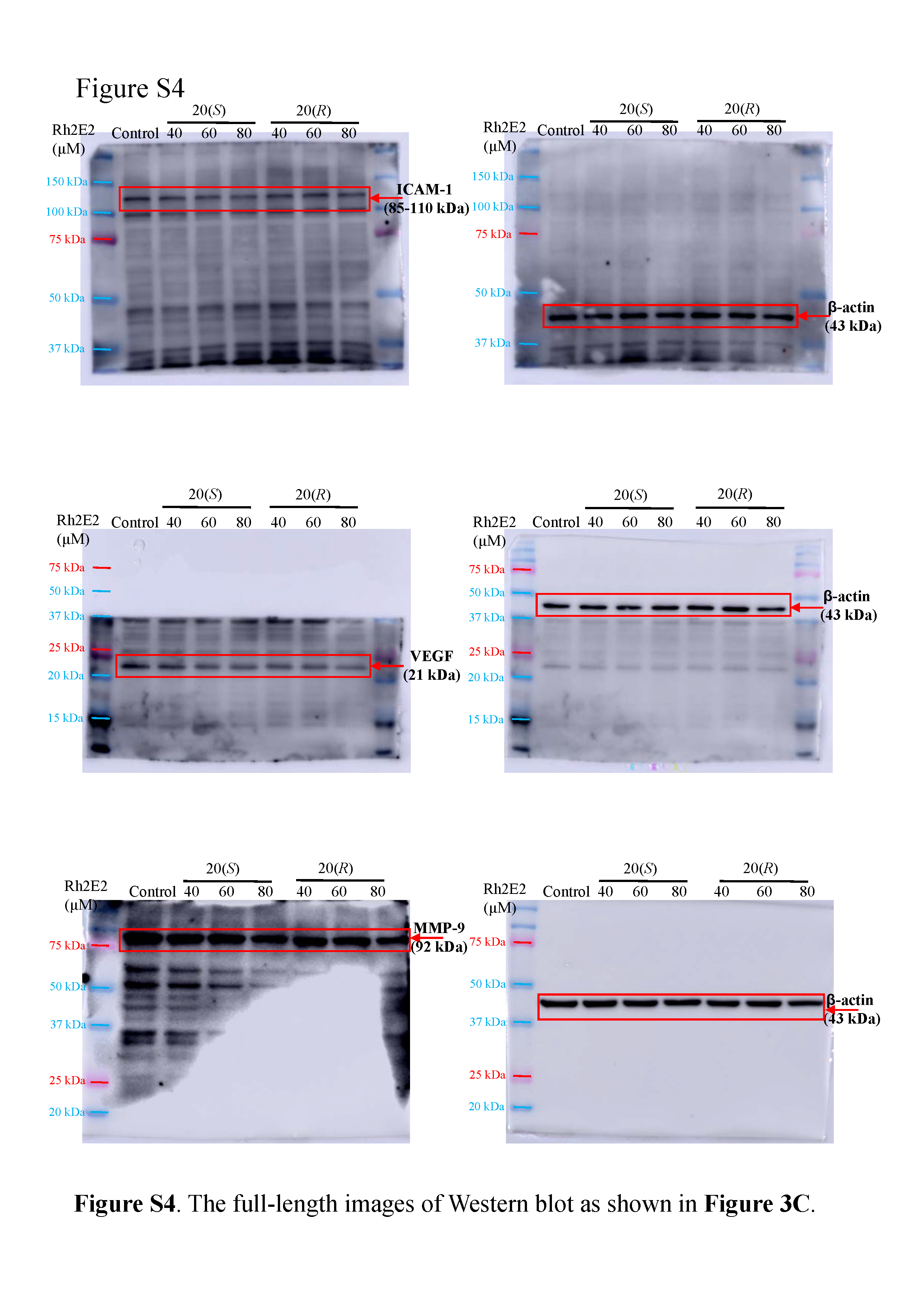

Supplement: Supplementary file 4 — Supplementary Figure S4 [file 41419_2020_2881_MOESM4_ESM.tif]

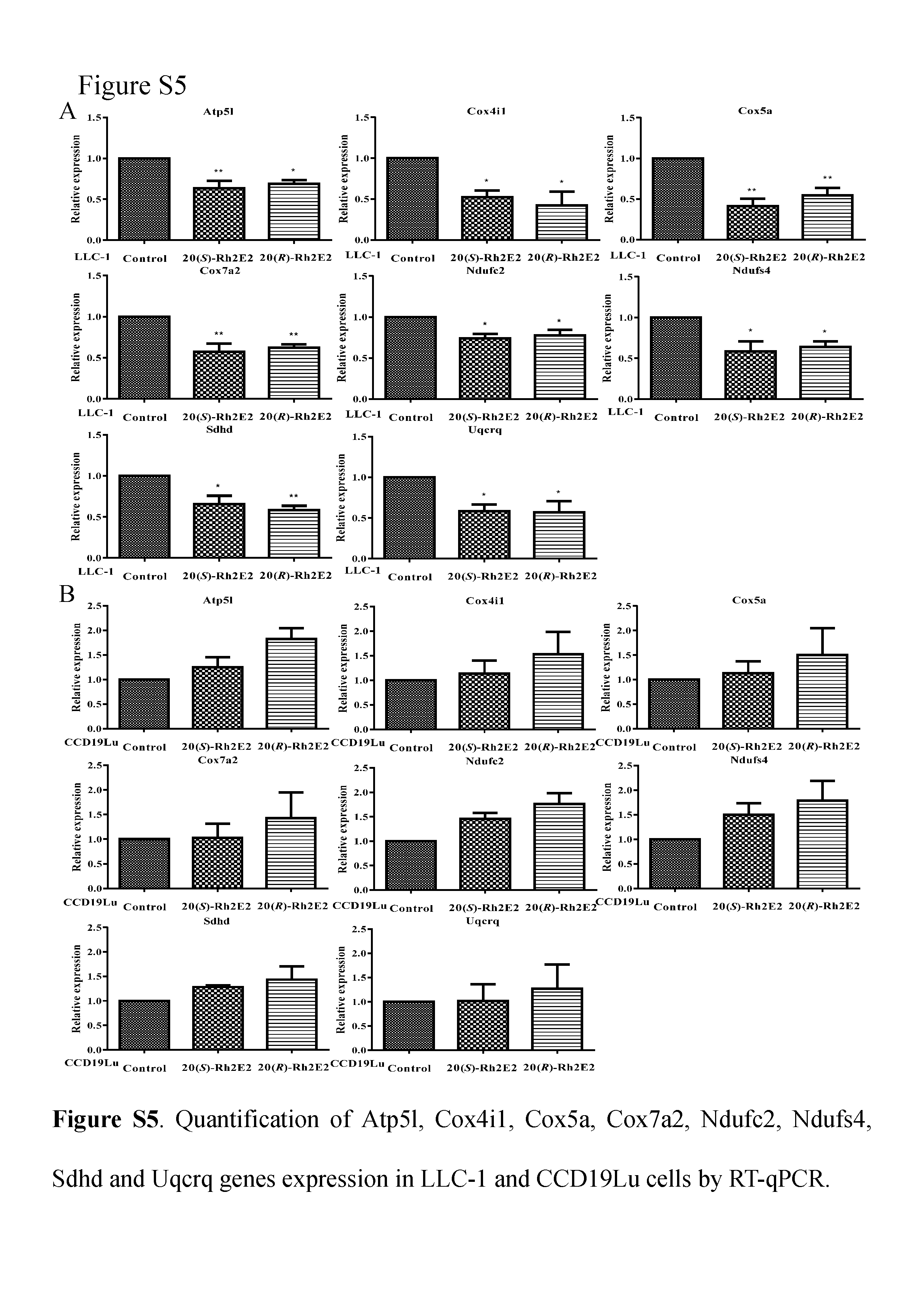

Supplement: Supplementary file 5 — Supplementary Figure S5 [file 41419_2020_2881_MOESM5_ESM.tif]

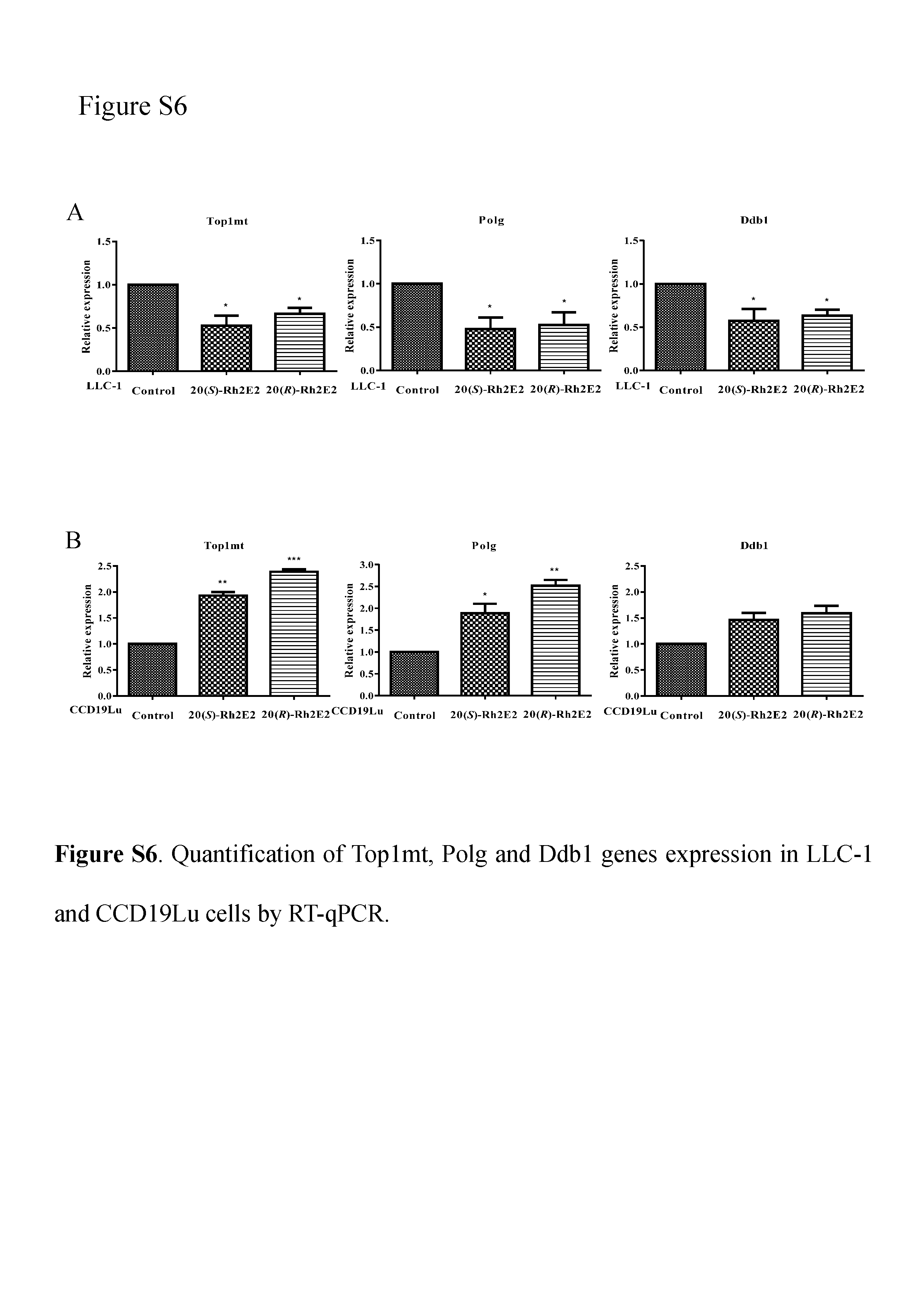

Supplement: Supplementary file 6 — Supplementary Figure S6 [file 41419_2020_2881_MOESM6_ESM.tif]

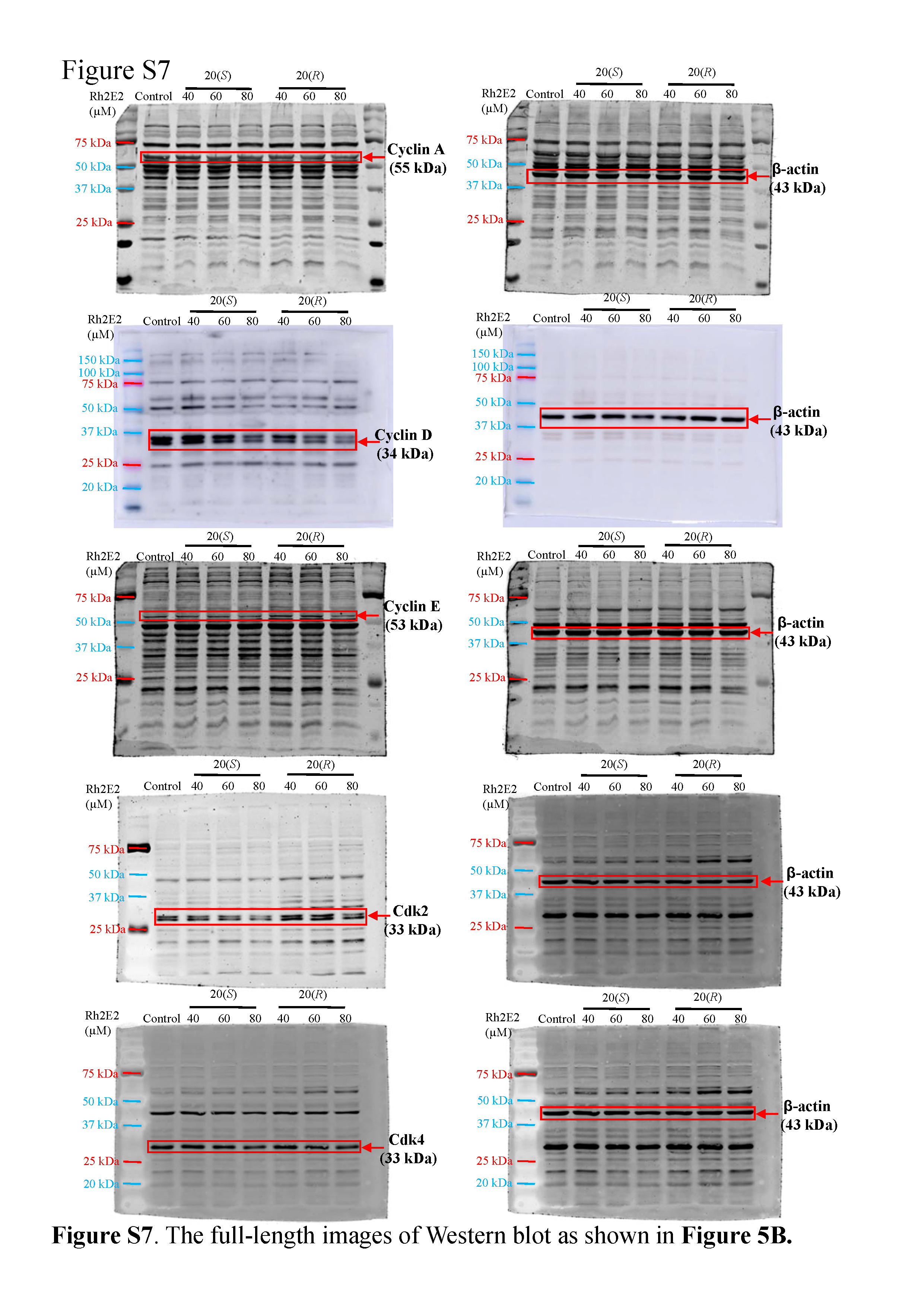

Supplement: Supplementary file 7 — Supplementary Figure S7 [file 41419_2020_2881_MOESM7_ESM.tif]

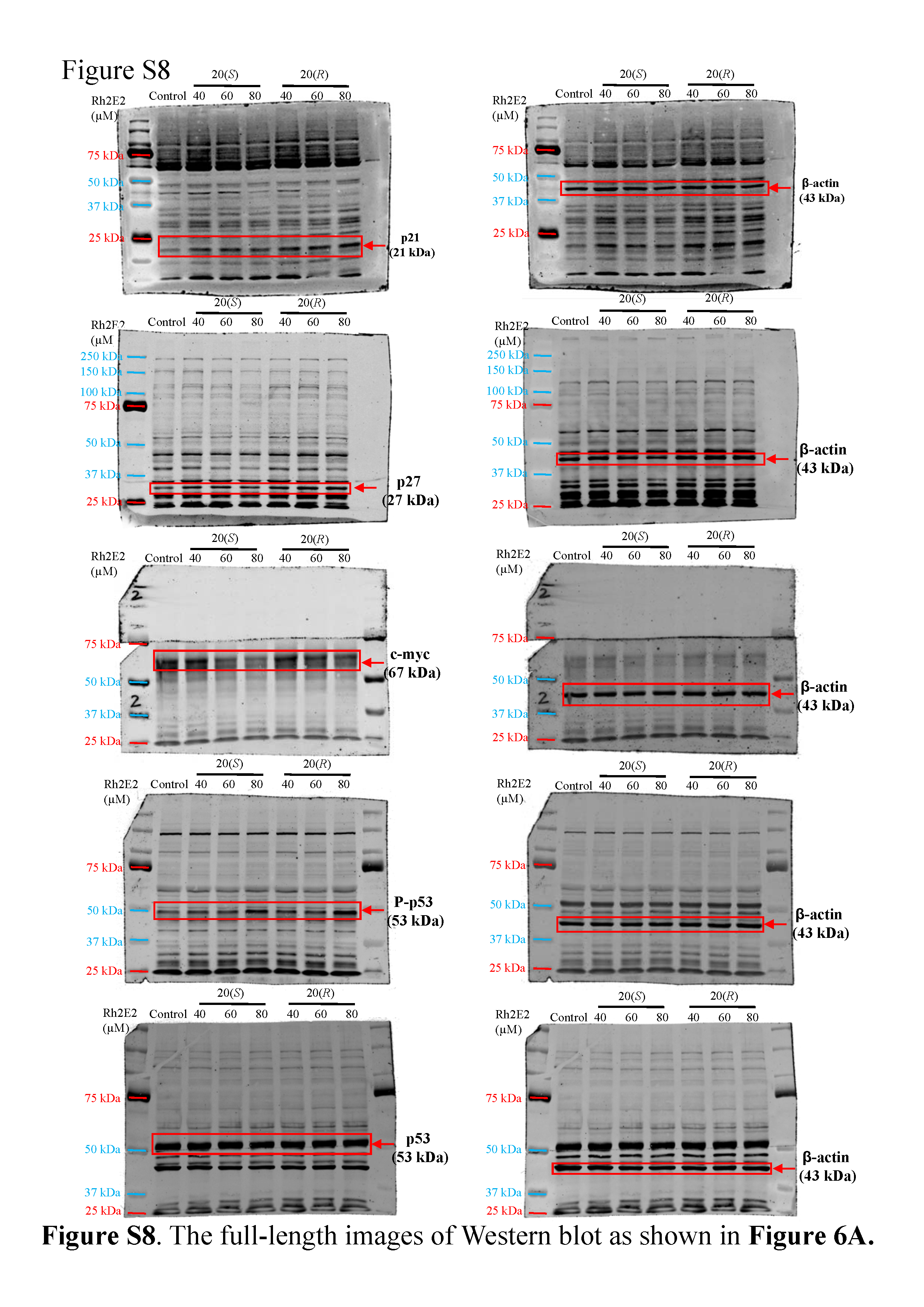

Supplement: Supplementary file 8 — Supplementary Figure S8 [file 41419_2020_2881_MOESM8_ESM.tif]

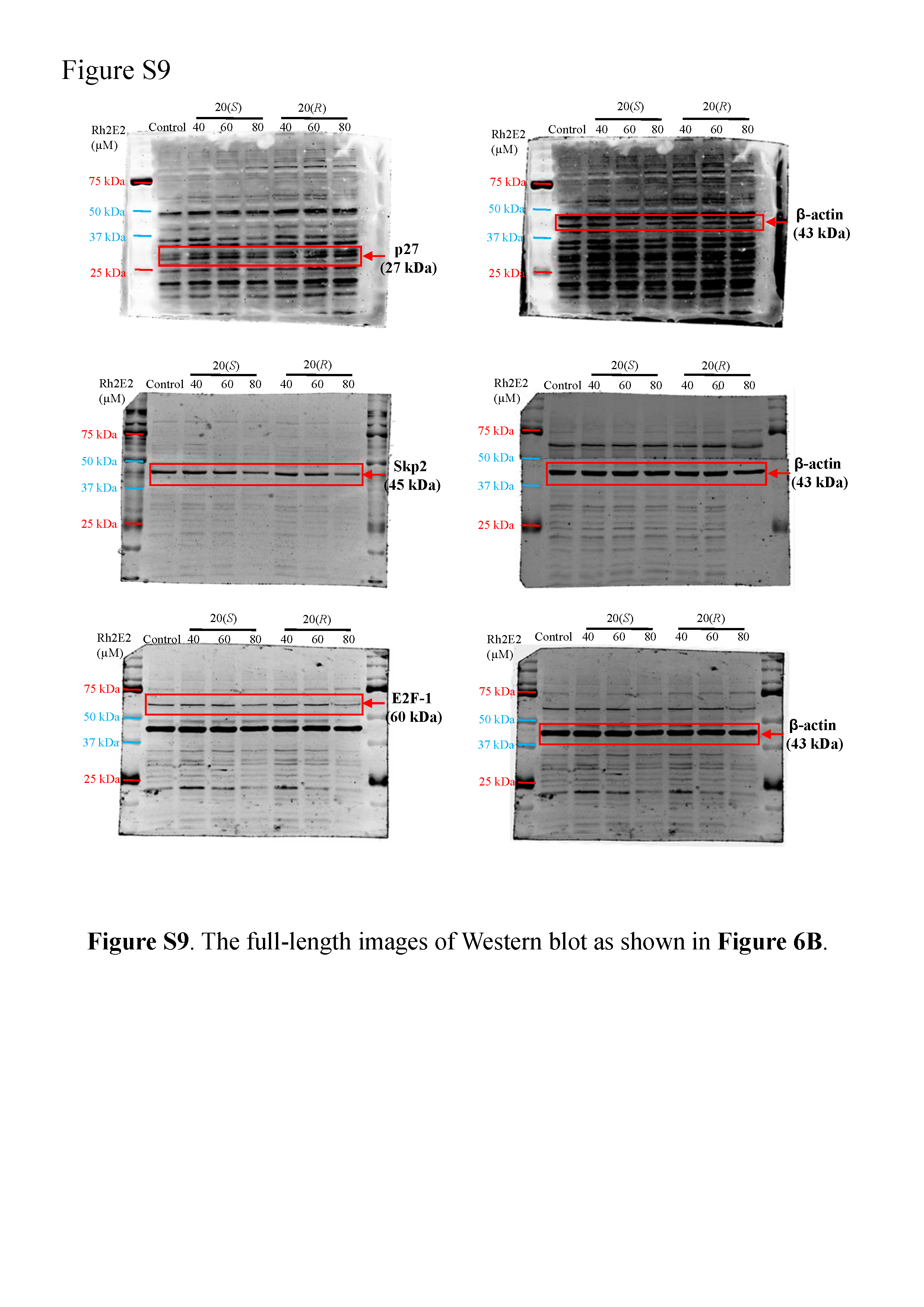

Supplement: Supplementary file 9 — Supplementary Figure S9 [file 41419_2020_2881_MOESM9_ESM.tif]

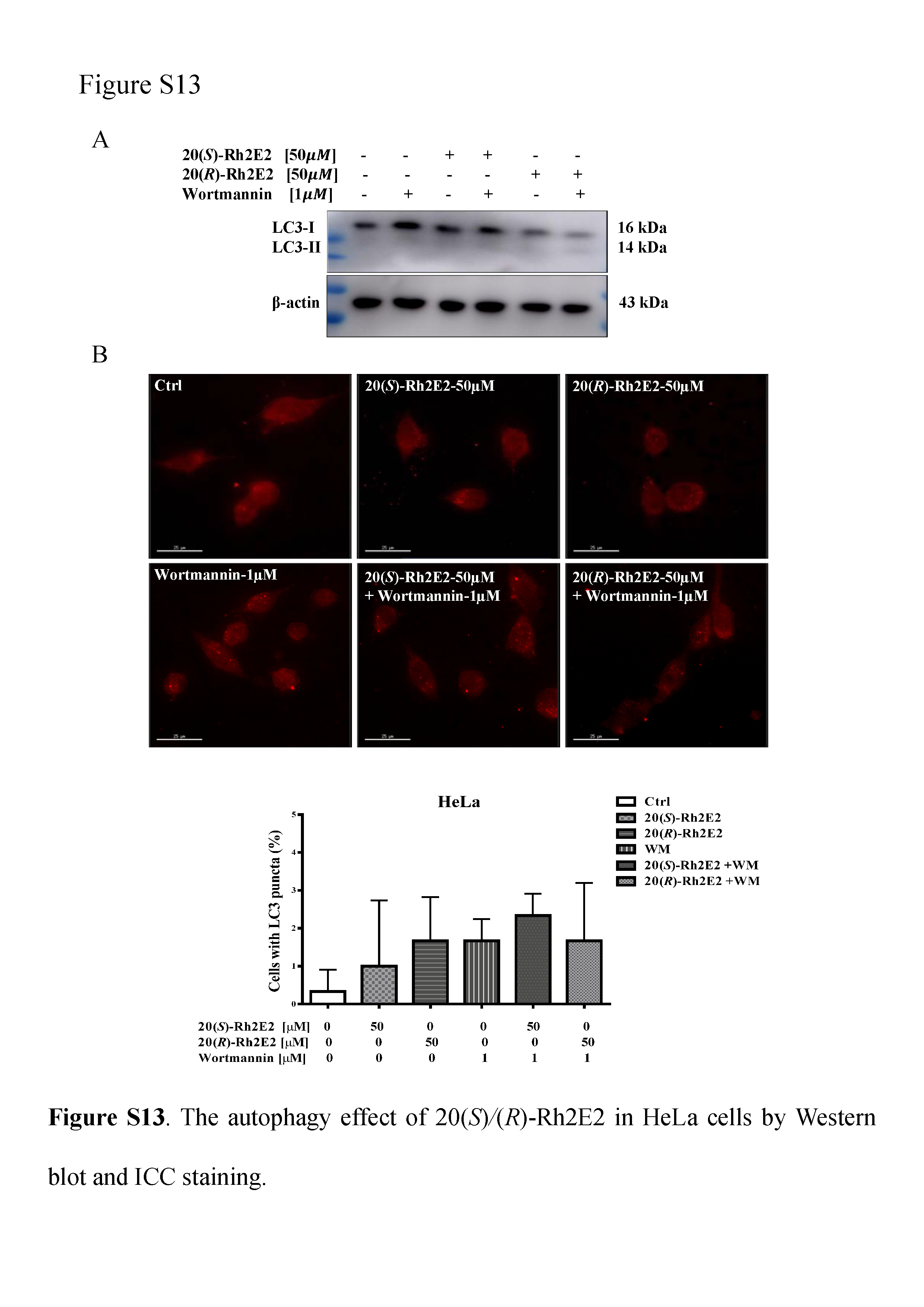

Supplement: Supplementary file 10 — Supplementary Figure S13 [file 41419_2020_2881_MOESM10_ESM.tif]

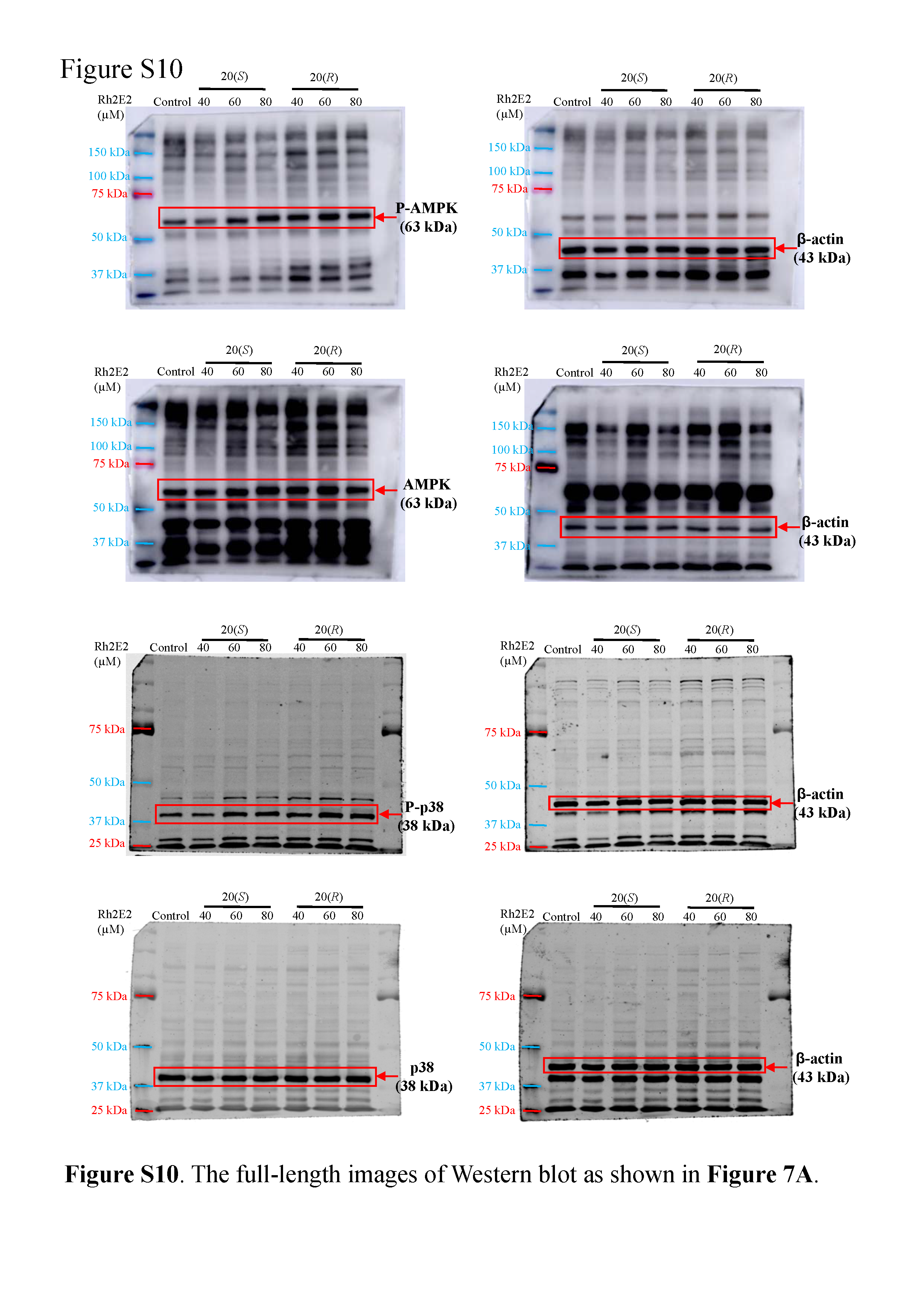

Supplement: Supplementary file 11 — Supplementary Figure S10 [file 41419_2020_2881_MOESM11_ESM.tif]

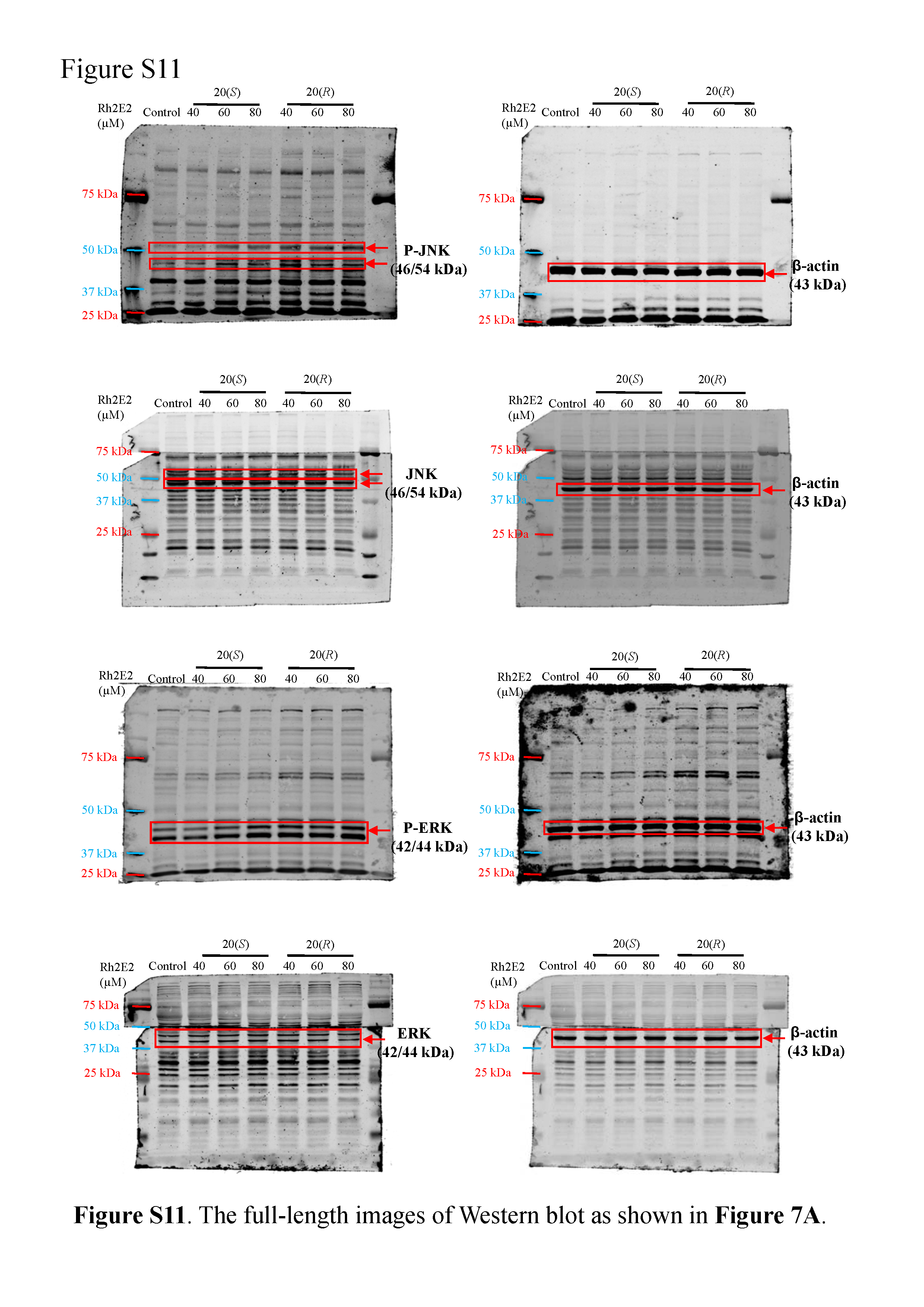

Supplement: Supplementary file 12 — Supplementary Figure S11 [file 41419_2020_2881_MOESM12_ESM.tif]

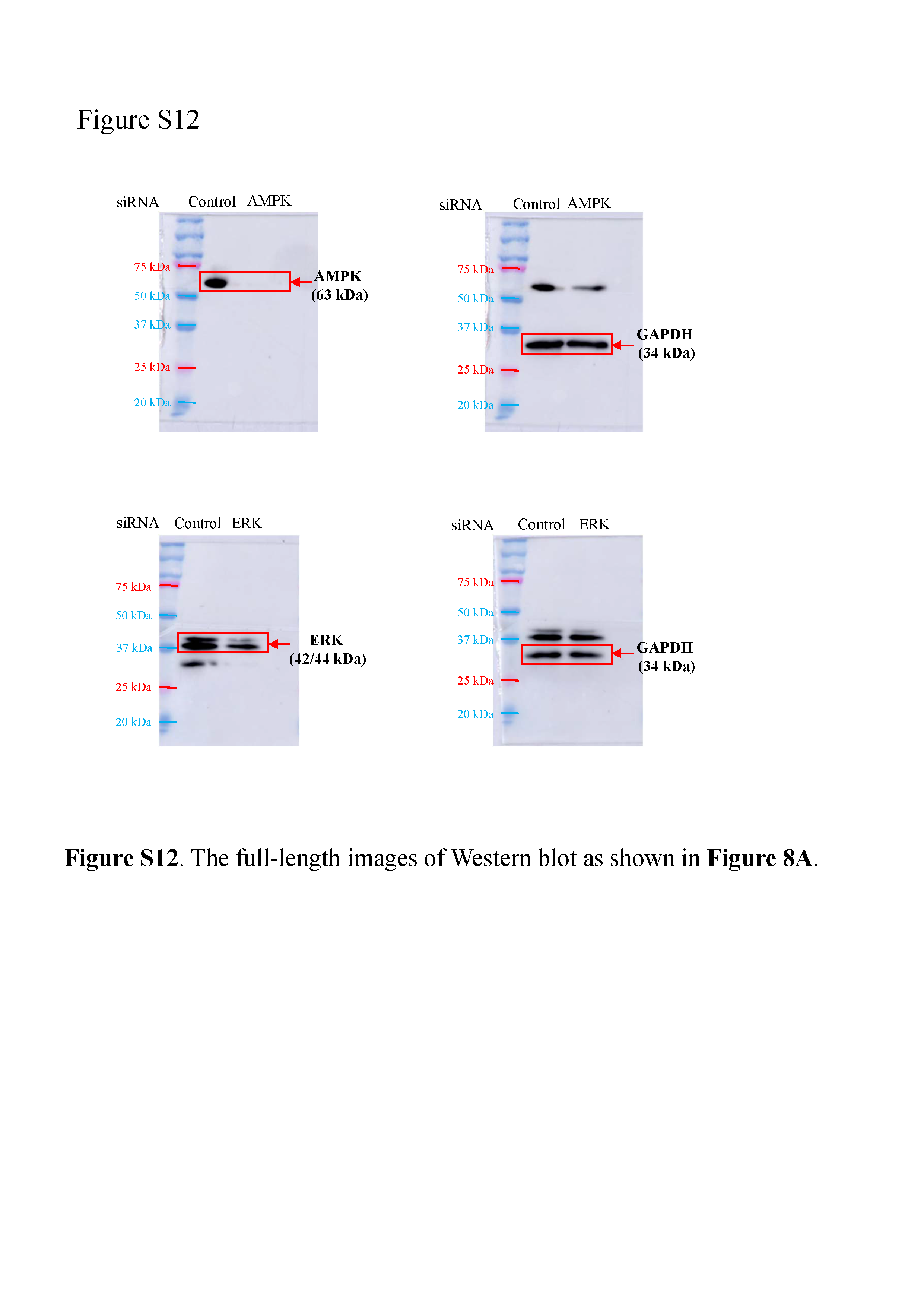

Supplement: Supplementary file 13 — Supplementary Figure S12 [file 41419_2020_2881_MOESM13_ESM.tif]
