# Supplementary material for: Novel ginsenoside derivative 20(S)-Rh2E2 suppresses tumor growth and metastasis in vivo and in vitro via intervention of cancer cell energy metabolism
Source: Cell Death Dis. 2020 Aug 14;11(8):621. doi: 10.1038/s41419-020-02881-4 (PMC7427995; doi:10.1038/s41419-020-02881-4)
Supplement: Supplementary file 14 — Supplementary Table S1 [file 41419_2020_2881_MOESM14_ESM.docx]

Table S1. RT-qPCR primer sequence of α-enolase and stathmin.

| H- Enolase -F | AGAAGTCCTGCAACTGCCTC |
| --- | --- |
| H- Enolase -R | ACCAGTCTTGATCTGCCCAGT |
| H- Stathmin -F | GATTCTCAGCCCTCGGTCAAA |
| H- Stathmin -R | TGCGTCTTTCTTCTGCAGCTT |
