# Supplementary material for: Novel ginsenoside derivative 20(S)-Rh2E2 suppresses tumor growth and metastasis in vivo and in vitro via intervention of cancer cell energy metabolism
Source: Cell Death Dis. 2020 Aug 14;11(8):621. doi: 10.1038/s41419-020-02881-4 (PMC7427995; doi:10.1038/s41419-020-02881-4)
Supplement: Supplementary file 15 — Supplementary Table S2 [file 41419_2020_2881_MOESM15_ESM.docx]

Table S2. RT-qPCR primer sequence of some enzymes belonging to oxidative phosphorylation.

| H- Atp5l -F | TGGTGAACGCTGCTGTGACT |
| --- | --- |
| H- Atp5l -R | CCCGCTTGCCTATAATCTCTCC |
| M- Atp5l -F | TAGCCGCTGCCGTGACTTACT |
| M- Atp5l -R | GCCACCAAACCATTCAGCACAG |
| H-Cox4i1-F | GCCCATGTCAAGCACCTGTCTG |
| H-Cox4i1-R | CCACAACCGTCTTCCACTCGTT |
| M-Cox4i1-F | ACGAGAGCTTCGCCGAGATGA |
| M-Cox4i1-R | TGTCCAGCATTCGCTTGGTCTG |
| H-Cox5a-F | GCCGTGGCTATCCAGTCAGTTC |
| H-Cox5a-R | GCCCGCAAAGCAGCATCAATG |
| M-Cox5a-F | CCGCCGCTGTCTGTTCCATT |
| M-Cox5a-R | ACCGTCTACATGCTCGCAATGC |
| H-Cox7a2-F | TGCGGAATCTGCTGGCTCTT |
| H-Cox7a2-R | GGCTCTATACAGGAGGGCATCA |
| M-Cox7a2-F | AGGACCATCAGCACCACTTCAC |
| M-Cox7a2-R | CCACCAAGCGTCAGAGCCATT |
| H-Ndufc2-F | CGGCTCCTCTACATCGGCTTCT |
| H-Ndufc2-R | TCACGGTCCCTCACAGCATACA |
| M-Ndufc2-F | GATGGACAACATGCTGCGGATG |
| M-Ndufc2-R | GCACTGGATGGAATGGCTCAAG |
| H-Ndufs4-F | GCGGCGGTGTCAATGTCAGT |
| H-Ndufs4-R | TTCCAGGCTTGGTGTGGCTAGA |
| M-Ndufs4-F | ATGGCGGCGGTCTCAATGTC |
| M-Ndufs4-R | TGTGCTCAACAACCTGGATGGA |
| H-Sdhd-F | ATGGCGGTTCTCTGGAGGCT |
| H-Sdhd-R | AATGGTGGCTCGGTGACAAGTG |
| M-Sdhd-F | CTGTCACCAAGCCACCACTCTG |
| M-Sdhd-R | AGCAAAGCCCAGCAAAGGTCAA |
| H-Uqcrq-F | AGCTACAGCTTGTCACCGTTCG |
| H-Uqcrq-R | ACACTACAAACTGCGGCACCAC |
| M-Uqcrq-F | CTCCTACAGCTTGTCGCCCTTT |
| M-Uqcrq-R | ACTGCTCAAACTCCTGGTTGCC |
