# Supplementary material for: Novel ginsenoside derivative 20(S)-Rh2E2 suppresses tumor growth and metastasis in vivo and in vitro via intervention of cancer cell energy metabolism
Source: Cell Death Dis. 2020 Aug 14;11(8):621. doi: 10.1038/s41419-020-02881-4 (PMC7427995; doi:10.1038/s41419-020-02881-4)
Supplement: Supplementary file 16 — Supplementary Table S3 [file 41419_2020_2881_MOESM16_ESM.docx]

Table S3. RT-qPCR primer sequence of mitochondria DNA level.

| H-Top1mt-F | CTACAACCGAGCCAACCGAGTC |
| --- | --- |
| H-Top1mt-R | GCCACCTGCTTGTTCTCCTCCT |
| M-Top1mt-F | GCGGACAGGCAATGAGAAGGAA |
| M-Top1mt-R | GCTTGTTCAGGCTGGAAGTGGT |
| H-Polg-F | GGTGTCTCCTACCTGCCTGTCA |
| H-Polg-R | GGCTGTGGCTGGTTCCTTCTTC |
| M-Polg-F | TATCGGCTGTCTGCGGATGGT |
| M-Polg-R | AACCTCGTCGTGGATGCTGATG |
| H-Ddb1-F | GGATGGCACCGTCACTCTCAAG |
| H-Ddb1-R | TCCAGGTCCACCACGCACAT |
| M-Ddb1-F | CAGTGCTGCTGCTTGCCTACA |
| M-Ddb1-R | TGCTGCCGCTCCTCATCAGT |
